# Unexpected Single-Ligand Occupancy and Negative Cooperativity in the SARS-CoV-2 Main Protease

**DOI:** 10.1021/acs.jcim.3c01497

**Published:** 2023-12-05

**Authors:** Simone Albani, Elisa Costanzi, Gia Linh Hoang, Maria Kuzikov, Marcus Frings, Narjes Ansari, Nicola Demitri, Toan T. Nguyen, Valerio Rizzi, Jörg B. Schulz, Carsten Bolm, Andrea Zaliani, Paolo Carloni, Paola Storici, Giulia Rossetti

**Affiliations:** † Institute for Neuroscience and Medicine (INM-9), Forschungszentrum Jülich, Jülich 52425, Germany; ‡ Faculty of Mathematics, Computer Science and Natural Sciences, RWTH Aachen, Aachen 52062, Germany; § Elettra–Sincrotrone Trieste S.C.p.A., SS 14 – km 163, 5 in AREA Science Park, 34149 Basovizza, Trieste, Italy; ∥ JARA-Brain Institute Molecular Neuroscience and Neuroimaging, Research Center Jülich GmbH, Jülich 52425, Germany; ⊥ RWTH Aachen University, Aachen 52056, Germany; # Fraunhofer Institute for Translational Medicine and Pharmacology (ITMP), Schnackenburgallee 114, Hamburg 22525, Germany; ∇ Fraunhofer Cluster of Excellence for Immune-Mediated Diseases (CIMD), Theodor Stern Kai 7, Frankfurt 60590, Germany; ○ Institute of Organic Chemistry, RWTH Aachen University, Landoltweg 1, Aachen 52074, Germany; ◆ Atomistic Simulations, Italian Institute of Technology, Via Enrico Melen, 83, 16152 Genova, Italy; ¶ Key Laboratory for Multiscale Simulation of Complex Systems, and Department of Theoretical Physics, Faculty of Physics, University of Science, Vietnam National University – Hanoi, 334 Nguyen Trai Street, Thanh Xuan, Hanoi 11400, Vietnam; ⋈ School of Pharmaceutical Sciences, University of Geneva, Rue Michel Servet 1, 1206 Genève, Switzerland; ⧓ Department of Neurology, Medical Faculty, RWTH Aachen University, Aachen 52074, Germany; ⧖ Jülich Supercomputing Center (JSC), Forschungszentrum Jülich, Jülich 52425, Germany; ∞ Constructor University, School of Science, Campus Ring 1, Bremen 28759, Germany

## Abstract

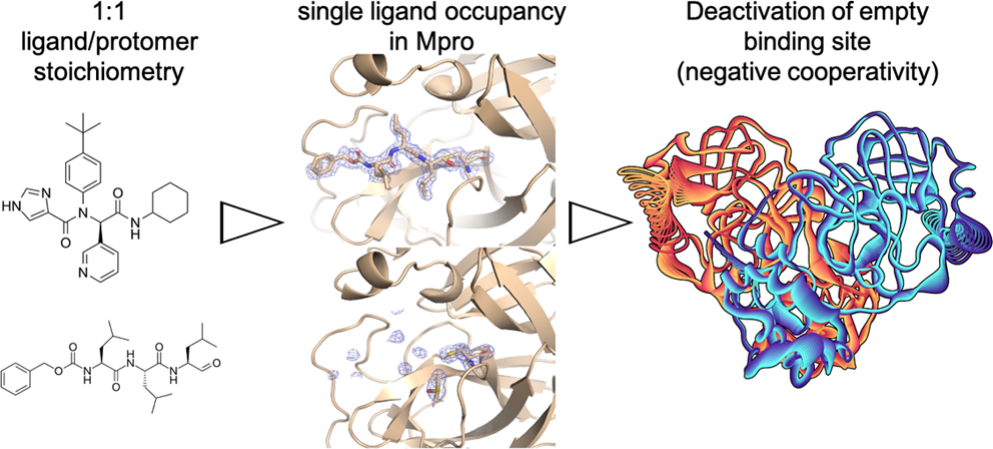

Many homodimeric enzymes tune their functions by exploiting either negative or positive cooperativity between subunits. In the SARS-CoV-2 Main protease (Mpro) homodimer, the latter has been suggested by symmetry in most of the 500 reported protease/ligand complex structures solved by macromolecular crystallography (MX). Here we apply the latter to both covalent and noncovalent ligands in complex with Mpro. Strikingly, our experiments show that the occupation of both active sites of the dimer originates from an excess of ligands. Indeed, cocrystals obtained using a 1:1 ligand/protomer stoichiometry lead to single occupation only. The empty binding site exhibits a catalytically inactive geometry in solution, as suggested by molecular dynamics simulations. Thus, Mpro operates through negative cooperativity with the asymmetric activity of the catalytic sites. This allows it to function with a wide range of substrate concentrations, making it resistant to saturation and potentially difficult to shut down, all properties advantageous for the virus’ adaptability and resistance.

## 1 Introduction

A significant fraction of enzymes are homodimers with one catalytic site in each subunit,^1^ active only in their dimeric states.^2−7^ This hints to an allosteric communication between the two sites and hence to cooperativity,^8^ which can be exploited for enzymatic function. The substrate affinity of a subunit upon substrate binding in the other one may increase (“positive cooperativity”, PC), thus increasing the enzymes’ sensitivity: a small change in ligand concentration gives rise to a large change in the concentration of the bound state of the protein.^9^ However, the allosteric interaction between subunits following the binding of the first ligand may also decrease the affinity for the second ligand into the other subunit (“negative cooperativity”, NC), allowing us to maintain enzymatic reactivity even in an excess of the substrate. This is a crucial feature for branching points in metabolic networks, which is the case where an intermediate species is chemically made or transformed by multiple enzymatic processes.^9,10^ Besides providing fundamental insights on enzymatic function, understanding the nature of cooperativity can help develop strategies for drug design.^11−14^

Several types of measurements have been used to investigate cooperativity in homodimeric enzymes: (i) detection of the occupancy status of ligands in the active sites: the presence of both subunits in apo or holo form hints to PC, while the presence of a ligand (substrate or inhibitor) only in one binding site suggests NC; (ii) the ligand input–output response measure: if a low ligand concentration leads to basically no output while a larger ligand content leads to almost maximal output, PC may be operative. However, if ligand depletion is considered, such response can also be characteristic of NC (especially when the ligand is appreciably depleted due to very high binding affinity).^15^ The situation is further complicated by the fact that NC cannot be distinguished from independent binding at multiple sites by equilibrium measurements.^16^ These two situations are not identical over the complete time courses of the binding reaction, but so far, the proposed approaches in pre-equilibrium conditions to distinguish between a NC model and a model where independent binding to multiple binding sites occurs can only evaluate how well the models fit the data, but not infer on the model itself.^16^ (iii) The value of the Hill Coefficient (HC), detected by input/output curves’ slopes: HC greater than 1 suggests PC, whereas HC lower than 1 hints to NC.^9,17,18^ However, this criterion has been criticized because (1) it assumes that ligands bind to the enzyme simultaneously,^18,19^ although ligand binding can alter the subunit-dimer equilibrium, becoming not simultaneous; (2) it does not consider the possibility that HC can be greater than 1 for covalent ligands, irrespectively of the nature of cooperativity.^20^ (iv) The symmetric nature of the homodimer structure: fully symmetric subunits may be characteristic of PC while asymmetric ones (both in the apo form and in the doubly occupied form) may be specific for NC.^9,21−24^ In both scenarios of cooperativity, symmetry plays a pivotal role. In the case of PC, the initial symmetry is disrupted upon the binding of the first ligand but is subsequently restored when the second catalytic site adopts a favorable conformation for binding. In the case of NC, the induced asymmetry is either maintained or amplified.^24^

**Figure 1:**
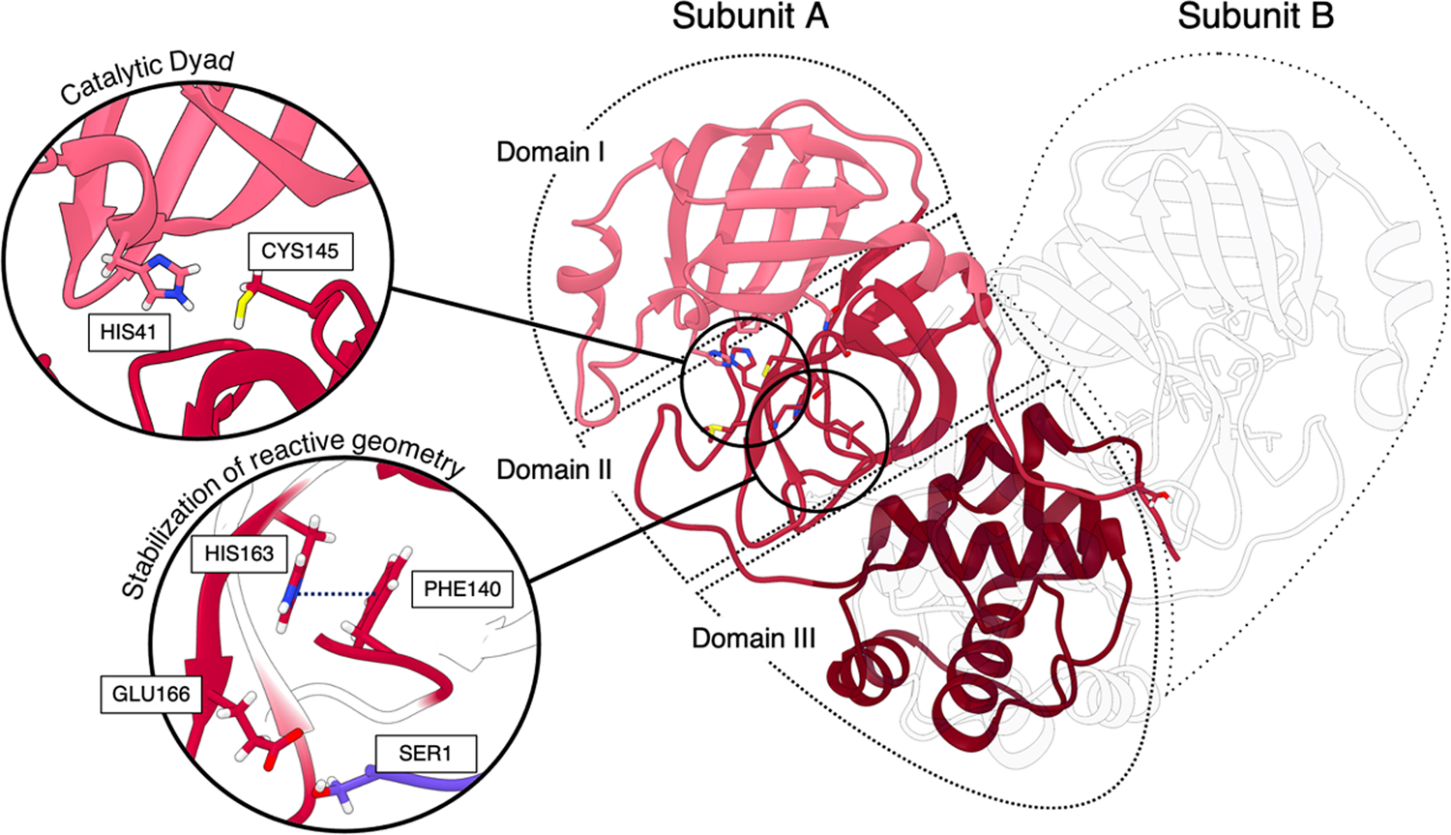
Ribbon representation of Mpro’s subunit “A”, shown in shades of red, and “B”, in the foreground, represented with gray low-opacity ribbons (PDBid 7PHZ). Each subunit consists of three domains. The first two are the chymotrypsin-like β-barrel domains I and II (residues 10–99 and 100–182, respectively) with six-stranded antiparallel β-barrels that harbor the substrate-binding site between them. The catalytic center is a CYS–HIS dyad. The last domain (residues 198–303) is a globular cluster of five helices involved in dimerization of the enzyme. The insets show details of the catalytic dyad and of the interactions that stabilize the reactive geometry and that were previously reported to be fundamental for site activation/deactivation;^32^ namely the hydrophobic interactions between PHE140 and HIS163, and the proximity of GLU166 to the SER1 of the adjacent protomer, which allows for the formation of interprotomer H-bonds.

From the discussion above, it is apparent that establishing unambiguously the nature of cooperation (especially NC) may be highly nontrivial. This is the case of the SARS-CoV-2 main protease (Mpro hereafter),^25,26^ a fundamental target against the virus.^27^ This enzyme is active only as a homodimer,^28^ with the N-finger of one monomer shaping the substrate-binding site of the other^26^ (Figure 1). This suggests a cooperation between the binding sites.^28^ However, the type of cooperativity has not been unambiguously demonstrated. On one hand, PC has been suggested by the following facts: (i) HC is greater than 1;^25,29,30^ however this could be caused by the fact that most of its ligands are covalent binders, as well as by the fact that ligand binding might not be simultaneous.^25^ (ii) Almost all of the ligand/protein complexes solved by macromolecular X-ray crystallography (MX) contain two ligands per dimer (as shown by an inspection of the 500 structures in the PDB Data Bank (https://www.rcsb.org), Table 1).^31^ (iii) The apo-Mpro and almost all (99%) of the ligand/Mpro complex MX structures exhibit dimeric symmetry. However, these facts could be the consequence of the excess of ligands added in the crystallization procedure (saturating both active sites), which might, in turn, cause the protein to crystallize as a homodimer with only one monomer in the asymmetric unit (see Section 2 for details).

**Table 1:** Ligand Binding and Symmetry in Previously Deposited Mpro Structures

ligands	covalent	symmetry	number	percentage
no	no	cyclic	186	26.7%
no	no	noncyclic	10	1.4%
yes	no	cyclic	163	23.4%
yes	no	asymmetric	4	0.6%
yes	yes	cyclic	328	47.1%
yes	yes	asymmetric	5	0.7%

On the other hand, NC could be suggested by observing that (i) some ligand/protein complex X-ray structures do exhibit asymmetry: namely, one subunit is not obtained by a symmetry operator on the other one, and the crystallographic unit contains the whole functional dimer(s). However, the overall number of such structures is very small (1.8%). (ii) A symmetry-breaking process of apo-Mpro occurs once it passes from the solid state to aqueous solution, as seen by long-time-scale molecular dynamics simulations.^32,33^ Such symmetry breaking has not been discussed for the doubly occupied enzyme. (iii) The enzymatic activity increases with the addition of catalytically inactive monomers in solution for SARS-CoV Mpro, which share 96% sequence identity.^34^ However, one has to be careful in drawing conclusions from one protein to the other, as they exhibit significant catalytic differences.^35^

To gain insight into the biophysics of this fascinating protein, here we attempt to establish the true nature of the enzyme’s cooperativity by applying an arsenal of biophysical methods. First, we ask ourselves whether the double occupancy might arise because an excess of ligand is used. We address this by using MX and binding assays. Next, we investigate the impact of solvation, which leads to a loss of symmetry of the apoprotein on passing from the solid state to aqueous solution.^32^ Anticipating our results, we show that in cocrystals obtained in conditions of 1:1 ligand/protomer stoichiometry, the protein features NC with only one ligand bound in one active site, possibly because binding of one ligand in the site distorts the other one. This contrasts with what was found in the 500 MX ligand-bound structures solved so far, which might have been obtained in an excess of ligands.

## 2 Experimental Section

### 2.1 Ligands

**MG-132** was purchased. **X77** was synthesized by us as follows. Rac-**X77** was prepared in two separate steps (Scheme S1). Although **X77** can be formed in only one step by the four-component Ugi reaction (Patent US9975885B2), we observed slightly higher yields when a preformed aldimine was utilized. Hence, the reaction of 3-pyridinecarboxaldehyde with 4-(*tert*-butyl)aniline in methanol at room temperature gave (*E*)-*N*-[4-(*tert*-butyl)phenyl]-1-(pyridin-3-yl)methanimine in quantitative yield. In the subsequent step, this aldimine was treated with 4-imidazole carboxylic acid and cyclohexyl isocyanide at 40 °C in methanol to furnish rac-**X77** in 45% yield after workup and purification (Figures S1–S9). Finally, **X77** and S-**X77** were successfully separated by preparative HPLC with a chiral stationary phase.

### 2.2 Biochemical Analyses of **X77**, S-**X77**, and Rac-**X77**

The SARS-CoV-2 Mpro was synthesized using the ORF1ab polyprotein residues 3264–3569 (GenBank code: MN908947.3). Gene synthesis, protein production, and purification were as reported by Zhang et al.,^26^ where eluted fractions containing the target protein were pooled and subjected to buffer exchange in 20 mM Tris-HCl, 150 mM NaCl, 1 mM EDTA, and 1 mM DTT, pH 7.8. The detection of enzymatic activity of the Mpro was performed under the conditions reported by Kuzikov et al.^36^

Enzymatic activity was measured by a Förster resonance energy transfer (FRET), using the dual-labeled substrate DABCYL-KTSAVLQ↓SGFRKM-EDANS (Bachem no. 4045664) containing a protease-specific cleavage site after the GLN. In the intact peptide, EDANS fluorescence is quenched by the DABCYL group. Following enzymatic cleavage, generation of the fluorescent product was monitored (Ex/Em = 340/460 nm) (EnVision, PerkinElmer). The assay buffer contained 20 mM Tris (pH 7.3), 100 mM NaCl, and 1 mM EDTA. The assay was established in an automated screening format (384-well black microplates, Corning, #3820) and optimized with respect to assay volume (10 μL), enzyme concentration (60 nM), substrate concentration (15 μM), incubation time (60 min with compounds, 15 min with substrate), temperature (37 °C for incubation with compounds, 25 °C for incubation with the substrate), DMSO tolerance (up to 5 v/v%), response to inhibition with known compounds such as zinc pyrithione, and the effects of reducing agents (DTT). **X77**, S-**X77**, and Rac-**X77** were then profiled in triplicate in 11 point concentration responses, starting from a 20 μM top concentration with 1:2 dilution steps.

### 2.3 X-ray Crystallography

#### 2.3.1 Crystallization

Crystallization of Mpro in complex with compounds was carried out as previously described.^37^ Briefly, Mpro, stored in 20 mM Tris-HCl, 150 mM NaCl, 1 mM EDTA, pH 7.8, and 1 mM DTT were incubated at 5 mg/mL (150 μM) with the compounds (**X77**/**MG-132**) at either 75 or 150 μM final concentrations. For **X77** also a 500 μM concentration was used. Crystallization experiments were set up after 1 h of incubation at RT, by seeding in sitting drops using the Morpheus kit (Molecular Dimensions) with a Mosquito robot (STPlabtech Ltd., Melbourn Hertfordshire, U.K.). Crystals appeared within a couple of days and were flash-frozen in liquid nitrogen after a few days of growth. For S-**X77** a 5 mM concentration of molecule was needed. For the “old” crystals, crystallization was carried out as described, with 5 mM **MG-132** or **X77**, respectively, and crystals were flash-frozen in liquid nitrogen after at least 2 months from their first appearance.

The best diffracting crystals appeared under the following conditions:

- Mpro:**X77** 500 μM, condition F10: 0.1 M Tris/BICINE pH 8.5; 0.12 M d-glucose; 0.12 M d-mannose; 0.12 M d-galactose; 0.12 M l-fucose; 0.12 M d-xylose; 0.12 M *N*-acetyl-d-glucosamine; 20% v/v ethylene glycol; 10% w/v PEG 8000.
- Mpro:**X77** 75 μM, condition H6: 0.1 M dl-glutamic acid monohydrate; 0.1 M dl-alanine; 0.1 M glycine; 0.1 M dl-lysine monohydrochloride; 0.1 M dl-serine; 0.1 M Hepes/MOPS pH 7.5; 20% v/v ethylene glycol; 10% w/v PEG 8000.
- Mpro:**X77** 150 μM, condition D6: 0.12 M 1,6-hexanediol; 0.12 M 1-butanol; 0.12 M 1,2-propanediol; 0.12 M 2-propanol; 0.12 M 1,4-butanediol; 0.12 M 1,3-propanediol; 0.1 M Hepes/MOPS pH 7.5; 20% v/v ethylene glycol; 10% w/v PEG 8000.
- Mpro:**MG-132** 75 μM, condition E2: 0.12 M diethylene glycol; 0.12 M triethylene glycol; 0.12 M tetraethylene glycol; 0.12 M penta-ethylene glycol; 0.1 M imidazole/MES pH 6.5; 20% v/v ethylene glycol; 10% w/v PEG 8000.
- Mpro:**MG-132** 150 μM, condition D1: 0.12 M 1,6-hexanediol; 0.12 M 1-butanol; 0.12 M 1,2-propanediol; 0.12 M 2-propanol; 0.12 M 1,4-butanediol; 0.12 M 1,3-propanediol; 0.1 M imidazole/MES pH 6.5; 20% v/v PEG 500 MME; 10% w/v PEG 20000.
- For Mpro:**X77** 500 μM enantiomer 1/R, condition D10: 0.12 M 1,6-hexanediol; 0.12 M 1-butanol; 0.12 M 1,2-propanediol; 0.12 M 2-propanol; 0.12 M 1,4-butanediol; 0.12 M 1,3-propanediol; 0.1 M Tris/Bicine pH 8.5; 20% v/v ethylene glycol; 10% w/v PEG 8000.
- For Mpro:**X77** 500 μM enantiomer 2/S and Mpro:**X77** 5 mM enantiomer 2/S, condition G4: 0.1 M sodium formate; 0.1 M ammonium acetate; 0.1 M sodium citrate tribasic dihydrate; 0.1 M potassium sodium tartrate tetrahydrate; 0.1 M sodium oxamate; 0.1 M imidazole/MES pH 6.5; 12.5% v/v MPD; 12.5% PEG 1000; 12.5% w/v PEG 3350.
- For Mpro:**X77** 5 mM enantiomer 1/R, condition E10: 0.12 M diethylene glycol; 0.12 M triethylene glycol; 0.12 M tetraethylene glycol; 0.12 M penta-ethylene glycol; 0.1 M Tris/bicine pH 8.5; 20% v/v ethylene glycol; 10% w/v PEG 8000.
- For Mpro:**MG-132** 5 mM “2-months old” crystal condition A2: 0.06 M magnesium chloride hexahydrate; 0.06 M calcium chloride dihydrate; 0.1 M Hepes/MOPS pH 7.5; 20% v/v PEG 500 MME; 10% w/v PEG 20000.
- For Mpro:**X77** 5 mM “2-months old” crystal condition G6: 0.1 M sodium formate; 0.1 M ammonium acetate; 0.1 M sodium citrate tribasic dihydrate; 0.1 M potassium sodium tartrate tetrahydrate; 0.1 M sodium oxamate; 0.1 M Hepes/MOPS pH 7.5; 20% v/v ethylene glycol; 10% w/v PEG 8000.

#### 2.3.2 Data Collection, Data Reduction, Structure Determination, Refinement, and Final Model Analysis

X-ray diffraction measurements were performed at 100 K at the XRD2 beamline of the Elettra synchrotron (Trieste, Italy) using a 1.000 Å wavelength. Crystals were flash-frozen in the original crystallization solution with no further addition of cryoprotectants. The collected data sets were processed using XDS^38^ and Aimless^39^ from the CCP4 suite.^40^

Structures were solved with Phaser^41^ by molecular replacement with 7BB2 (PDBid) as a search model. Refinement was carried out by alternating cycles of manual model building in COOT^42,43^ and automatic refinement using Phenix^44^ (version 1.19.2_4158) is reported in Table S1. Figures were prepared using Pymol.^45^

#### 2.3.3 Data Availability

Coordinates and structure factors were deposited in the Protein Data Bank with accession numbers 7PHZ (Mpro:**X77** in space group P212121), 8P57 (Mpro:**X77** at 75 μM), 8P56 (Mpro:**X77** at 150 μM), 8P55 (Mpro:**MG-132** at 75 μM), 8P54 (Mpro:**MG-132** at 150 μM), 8P58 (Mpro:R-**X77** at 500 μM), 8P5A (Mpro:R-**X77** at 5 mM), 8P5B (Mpro:S-**X77** at 500 μM), 8P5C (Mpro:S-**X77** at 5 mM), 8P86 (Mpro:**MG-132** at 5 mM, “2-months-old” crystal), and 8P87 (Mpro:**X77** at 5 mM, “2-months-old” crystal). PDB X-ray structure validation reports of the deposited structures can be found in the Supporting Information: “Full wwPDB X-ray Structure Validation Report”.

#### 2.3.4 Analysis of Previously Deposited Structures

A tabular report and corresponding structures were downloaded for 696 SARS-CoV-2 Mpro entries, deposited in the PDB database between 5th February 2020 and 26th April 2023. Among these, 497 structures were found to contain nonsolvent ligands with molecular weight ≥100 Da. An in-house python script was used to check for covalent bonds between protein and ligands, which were present in 333 structures out of 497. Percentages reported in the text are derived from the results summarized in Table 1. The classification of structures in this table has been manually curated. For instance, a structure in which Mpro active site interacts with another protein classified as “apo” required a manual correction. Additionally, some of the submitted structures might not contain all domains of Mpro, i.e., six asymmetric structures with a ligand do not contain the whole dimer within the unit cell. Among the 10 apo structures with noncyclic symmetry, the distribution of space groups is the following: P1: 3,P2_1_ 2_1_ 2_1_: 2, P12_1_1: 2, *P*2_1_2_1_2: 2, *P*4_3_2_1_2: 1.

### 2.4 Simulations

#### 2.4.1 Molecular Dynamics

The systems 6W63, 7PHZ, and 8P57 were studied in 500-ns unbiased MD simulations, using GROMACS 2019.2^46^ and the Amber14SB force field.^47^ The TIP3P model was used for the water molecules, while the ligand was parameterized using the General AMBER Force Field (GAFF) with AM1-BCC charges.^48^ The protein was preprocessed using Schrodinger’s Protein Preparation Wizard^49^ and the protonation state of residues in the active site was compared and confirmed with the output of the VirginiaTech H++ Web Server.^50^ N-terminal acetyl and C-terminal amide capping groups were added to the 7PHZ and 8P57 structures. The protein and the ligand were then placed at the center of a 16 × 16 × 16 cubic nanometers box and solvated with water and 0.15 M NaCl. The systems were minimized with 50,000 steps of steepest descent and 50,000 steps of conjugate gradient and then heated from 5 to 310 K over the course of 5 ns, followed by a 1 ns equilibration stage in an NPT ensemble. During the annealing and NPT equilibration, 1000 kJ/mol restraints were applied on the C α atoms and on the ligand, along all three coordinates. The restraints were then released for the 500-ns unbiased simulation conducted with a time step of 2 fs, Parrinello-Rahman barostat, Velocity Rescale thermostat, and LINCS constraints on all bonds. Long-range electrostatics interactions were handled with Particle Mesh Ewald (PME) using 1.6 Å grid spacing. The cutoff radius of van der Waals interaction and short-range electrostatics was set to 1.2 Å.

#### 2.4.2 Water Analysis

The analysis was conducted using an in-house python (v 3.10.6)^51^ script with the packages MDtraj (v 1.9.7)^52^ and alphashape (v 1.3.1).^53^ We investigated the change in the number of water molecules within the region around the binding pocket S1 during MD simulations. This region was defined by a convex hull bordered by the α carbon atoms (CA) of residues VAL114, ALA116, GLY138, PHE140, ASN142, GLY146, HIS164, HIS172, and GLY174, the carbonyl carbon atom of residue Thr135, and the carbonyl oxygen of residue CYS117. The analysis was performed on 5000 frames of a 500-ns MD trajectory for each system.

#### 2.4.3 Principal Component Analysis

The analysis was performed on the last 400 ns of simulation time, with a sampling time step of 0.1 ns. The two subunits in each simulation were analyzed separately and only the α carbon atoms were considered. The standard GROMACS tools gmx covar and gmx anaeig were used for the analysis and for the generation of the protein structures deformed along the first eigenvector.

## 3 Results and Discussion

### 3.1 Macromolecular Crystallography and Binding Essays

Using nonsaturating conditions, namely 1:1 and 1:2 ligand/monomer stoichiometries (LMS), we solved 4 new MX structures to be added to the ∼500 already deposited Mpro/ligand complex structures, which were possibly all determined in excess of ligand and almost in their entirety, exhibiting a double occupancy of the ligand.

**Figure 2:**
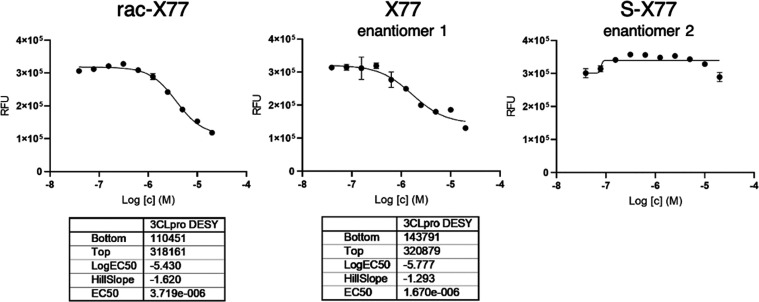
Dose–response curves for rac-**X77**, **X77**, and S-**X77** and in the biochemical assay for Mpro. S-**X77** does not exert any inhibitory activity Mpro.

**Chart 1:**
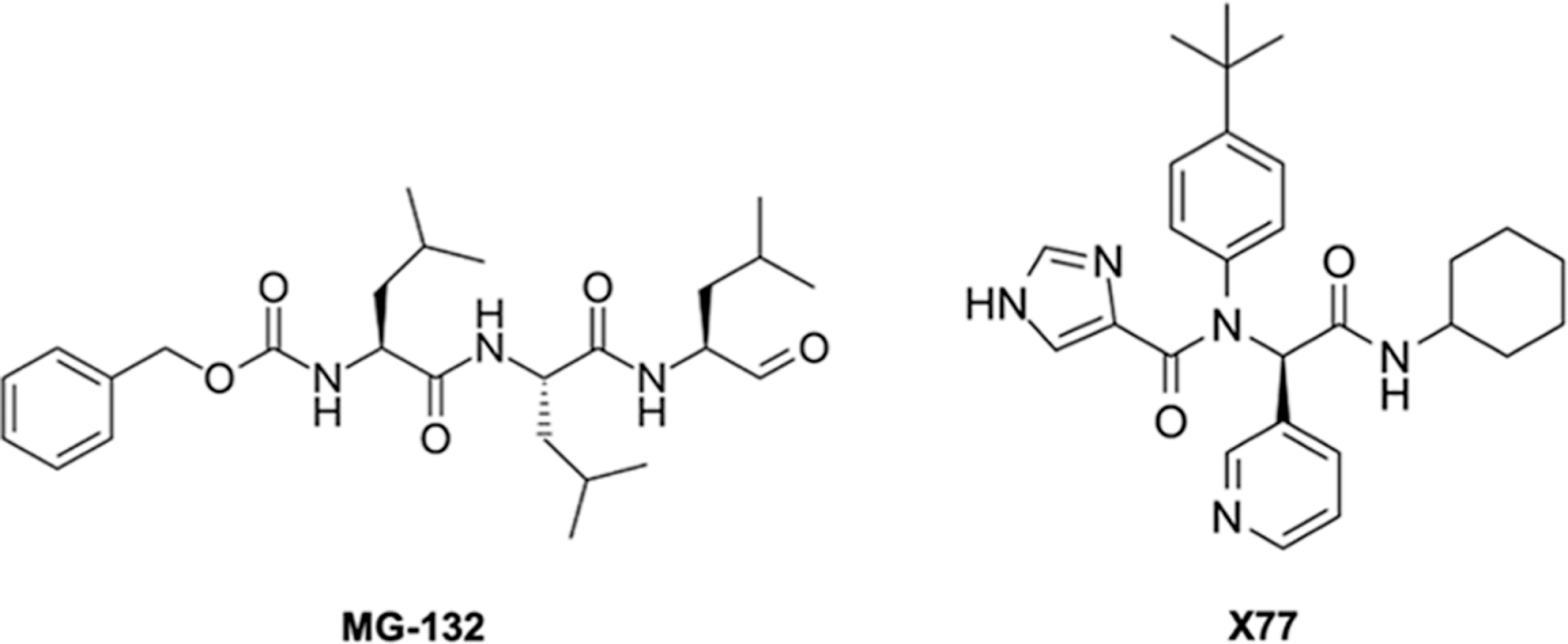
Chemical Structure of Cocrystallized Ligands

The first ligand is benzyl *N*-[(2*S*)-4-methyl-1-[[(2*S*)-4-methyl-1-[[(2*S*)-4-methyl-1-oxopentan-2-yl]amino]-1-oxopentan-2-yl]amino]-1-oxopentan-2-yl]carbamate, **MG-132**, in Chart 1, which forms a covalent bond with CYS145, and its IC50 for MPro is 7.4 μM.^36,37^ The MX structure bound to **MG-132** with double occupancy was solved previously by some of us at 1.94 Å in the C2 space group (PDBid 7NF5) and also at 1.68 Å resolution in the P212121 space group (PDBid 7BE7), in the condition of excess of ligands.^37^ The second ligand is the R-enantiomer *N*-(4-tert-butylphenyl)-*N*-[(1*R*)-2-(cyclohexylamino)-2-oxo-1-(pyridin-3-yl)ethyl]-1*H*-imidazole-4-carboxamide, **X77** in Chart 1, which forms only noncovalent interactions. The MX structure with double-ligand occupation was reported at 2.1 Å in the C2 space group (PDBid: 6W63) and we reproduced it in our crystallization condition in space group P212121 (PDBid: 7PHZ). Its inhibitory activity for Mpro, along with that of the S-enantiomer (S**-X77** hereafter) and that of the racemate (rac-**X77**), was not known when we started this study. They were measured here employing a Förster resonance energy transfer (FRET) with a dual-labeled substrate, DABCYL-KTSAVLQ↓SGFRKM-EDANS (Bachem #4045664), containing a protease-specific cleavage site after the GLN. In the intact peptide, EDANS fluorescence is quenched by the DABCYL group. Its inhibitory activities are reported in Figure 2 as dose–response curves. **X77** and S-**X77** were identified by a comparison with X-ray experiments, where the two enantiomers were separately cocrystallized with Mpro, solving 4 crystal structures, with the two enantiomers at two different concentrations (see the below paragraph). The inhibitory activities are reported in Figure 2 as dose–response curves. The racemate showed an IC50 of 3.7 μM, while that of **X77** is 1.7 μM. The S-**X77** curve could not allow IC50 calculation, as no real dose–response could be measured: likely, this enantiomer could not properly bind to stop the reaction. Indeed, this was confirmed by solving the crystal structure with the S-**X77** enantiomer (see the below paragraph).

### 3.2 Complex with **MG-132**

**Figure 3:**
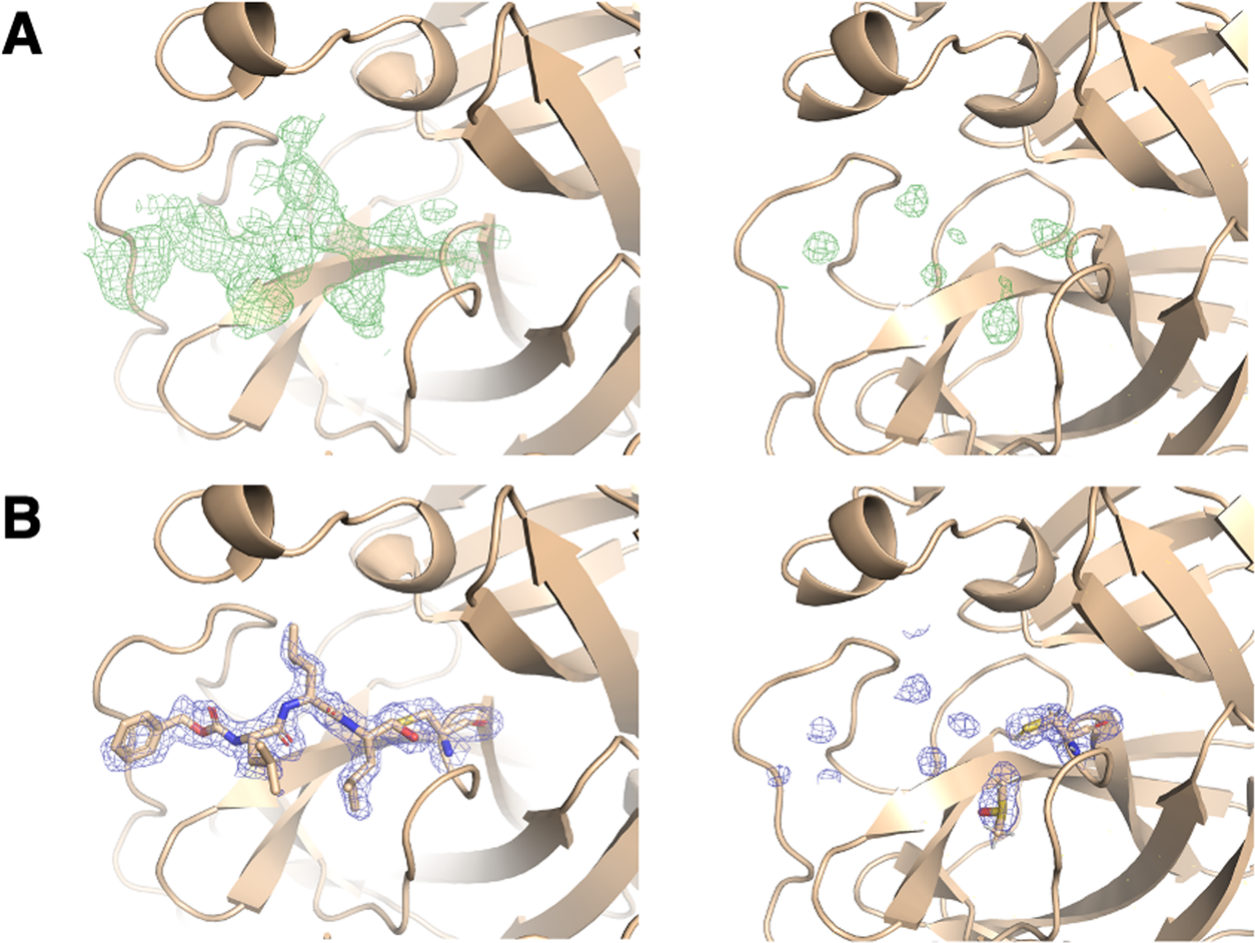
**MG-132** complex: (A) Initial Fo–Fc maps contoured at 3 sigma for chain “A” (right) and chain “B” (left) of the complex obtained with a ligand/protein ratio of 1:1 (PDBid 8P55). (B) Final 2Fo–Fc maps contoured at 1 sigma for chain A (right) and chain B (left) of 8P55 (i.e., 75 μM of **MG-132**). Polder omit maps of the ligand placed in both chains were generated and confirm the results observed in the initial Fo–Fc difference maps (Figure S12). **MG-132** is covalently bound to the sulfur atom of catalytic CYS145. The nitrogen atoms of the backbone of this peptidic ligand act as the hydrogen bond donor toward the residues HIS164 backbone and GLN189 side chain. The last carboxyl and amide groups in the ligand’s backbone form two additional hydrogen bonds with the backbone of GLU166. The terminal benzyl group is stabilized by hydrophobic contacts with the C atoms in the side chains of LEU167, PRO168, and GLN192 (Figure 4B).

The 150 μM protein solution was incubated with the **MG-132** inhibitor in nonsaturating conditions, namely 1:1 and 1:2 LMS, following our standard protocol to obtain crystals in space group P212121 with the entire dimer/au. The two binding sites of our resulting crystal structures, solved at 1.85 and 1.60 Å resolution respectively (PDBid: 8P55 and 8P54), showed clear dissimilarities: The difference electron density map of one subunit showed a continuous positive electron density that well fit the **MG-132** moiety, while in the other subunit no residual electron density was present, suggesting an empty pocket (Figure 3A). Even after refinement, no further density appeared in the second binding site (Figure 3B). This establishes a single occupancy of the ligand.

**Figure 4:**
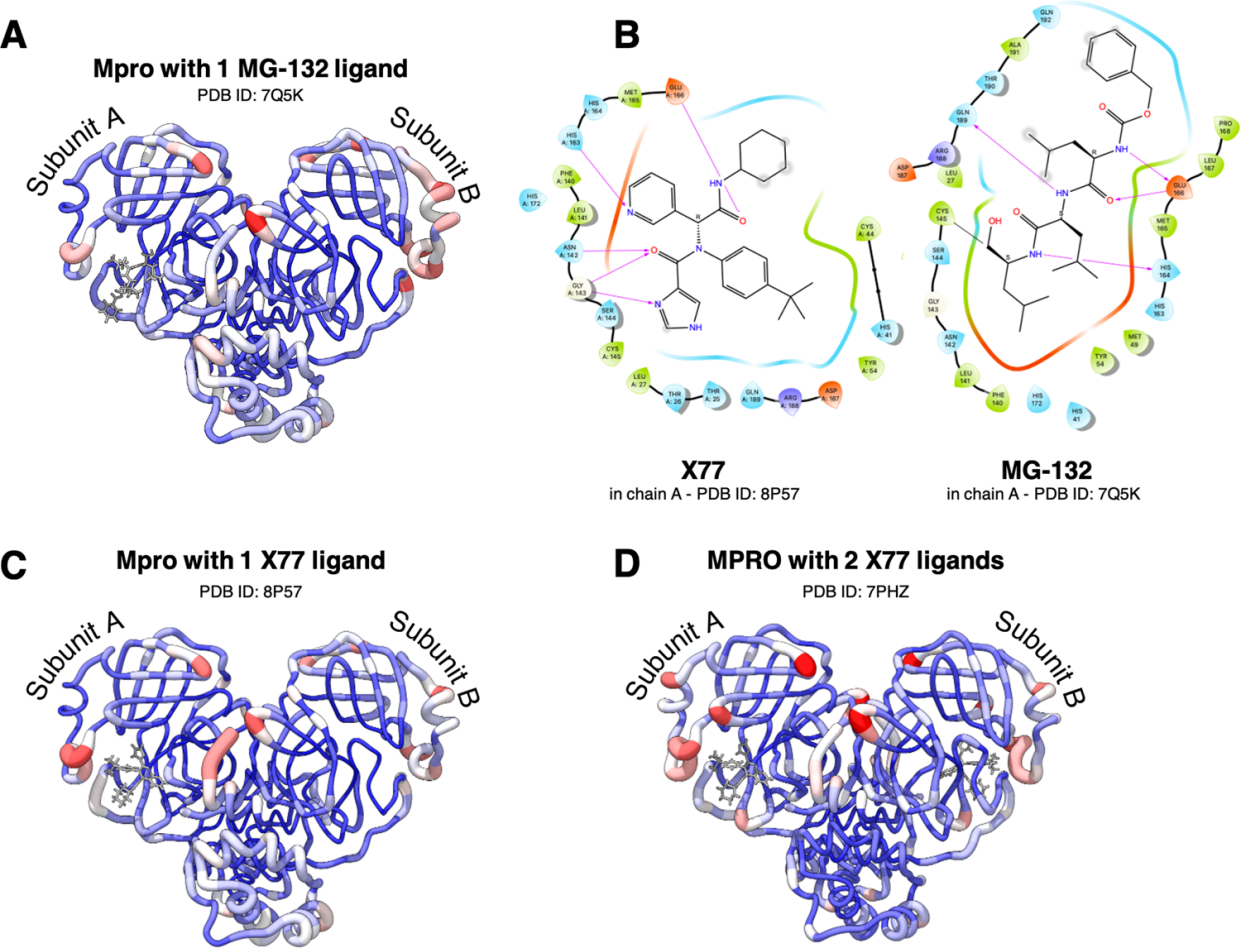
B-factors and binding modes in MX. (A) B-factor ‘cartoon putty’ representation of 8P55 (in each dimer, left = subunit A, right = subunit B). The pink to red colors and a wider tube indicate regions with higher B-factors, whereas shades of blue and a narrower tube indicate regions with lower B-factors. (B) 2D schematic representation of the interactions between Mpro and the ligands **X77** and **MG-132**, as observed in the PDB structures with ID 8P57 and 8P55. Residue color legend: light blue = polar, red = negative, violet = positive, green = hydrophobic, light gray = glycine. ‘Cartoon putty’ representation of the B-factors of structures (C) 8P57 and (D) 7PHZ.

The binding pose of the ligand is the same as that observed in the doubly occupied enzyme previously solved^37^ (adduct root-mean-square deviation of 0.75 and 0.32 Å with 7NF5 chain “A” and 7BE7 chain “A”, respectively). The b-factors of chain “B” (not containing the ligand) are larger than those of “A” (Figures 4A and S10).

A fully consistent picture is obtained by letting the double-occupied crystal for 2 months in their growing solution: cocrystals of Mpro obtained in excess of **MG-132** as described in ref (37) after 2 months changed, showing a positive Fo–Fc difference map corresponding to the covalently bound ligand only in one chain, while the other resulted empty (Figure S11). The single-occupied site crystals diffract to resolutions similar to those of fresh crystals, around 1.85 Å. This shows that only one binding site remains occupied if the enzyme is allowed enough time to allow one ligand to break its covalent bond and diffuse. The results strikingly differ from freshly obtained crystals prepared with the same protocol, which clearly showed to have both sites occupied.^37^

### 3.3 Complex with **X77**

The MX structure was solved in nonsaturating conditions (again 1:1 and 1:2 LMS, PDBid: 8P56 and 8P57), at a resolution ranging from 1.85 to 1.60 Å (Table S1) and in excess of ligand (PDBid: 7PHZ). Note that all of the structure crystallizes in the same P212121 space group (with the entire dimer/au). As observed for **MG-132**, the ligand occupies only one active site in nonsaturating conditions (Figure 4C). The presence of the empty cavity is evident by the Fourier difference map Fo–Fc, with reduced mobility in the ligand-bound subunit, again emerging by the values of the b-factors (Figure 4C). The ligand occupies both sites when in excess (Figure 4D) as it does in the reported X-ray structure (PDBid 6W63). However, also in this case, the *b*-factors of chain B are higher (Figures 4D and S10). As in the above case, the pose is the same as that of the structures in excess of the ligand observed by others (PDBid 6W63) or here (PDBid 7PHZ) (Figure S13). In detail, **X77** carboxyl moieties accept hydrogen bonds from the backbone of the protein through residues GLU166 and GLY143. The former residue can establish a hydrogen bond with the imidazole N_ε_ atom of **X77**. Also, the pyridyl ring is stabilized by a hydrogen bond, in this case with the side chain of HIS163. Additionally, water-mediated hydrogen bonds further contribute to the stability of the molecule (e.g., interaction between imidazole N_γ_ and the HIS41 backbone) (Figure 4B). Notably, as in the **MG-132** case, when the crystals obtained in excess of the ligand are left for 2 months in their crystallization solution before being flash-frozen for the diffraction experiments, the latter showed unambiguously only one occupied cavity, demonstrating that **X77** remained bound at one site while diffused from the other one (PDBid: 8P87) (Figure S11).

### 3.4 Complex with S-**X77**

We obtained crystal structures in the presence of the two enantiomers, respectively, at resolution 1.55 Å for enantiomer 1 and at resolution 1.47 Å for enantiomer 2. As shown in Figure S14A, we could prove that enantiomer 1 had the R configuration by the unambiguous electron density reproducing the result obtained with the racemic mixture. In the crystal structure obtained in the presence of enantiomer 2, instead we saw small blobs of electron density that could be modeled with a DMSO and water molecules (Figure S14B). With the refinement of the structure obtained in the presence of enantiomer S, small positive blobs of not modellable Fo–Fc were left. We repeated the crystallization experiments of both enantiomers using the highest reachable concentration, taking into account the DMSO tolerance of the protein. Crystallization trials were set up in the presence of a 5 mM inhibitor, and the crystals diffracted at resolutions of 1.66 Å for enantiomer 1/R and 1.51 Å for enantiomer 2/S. For enantiomer 1/R the results reproduced the same results as for lower concentrations (Figure S14C). Interestingly, for enantiomer 2/S, we obtained a positive Fo–Fc density that allowed the modeling of the enantiomer, as shown in Figure S14D. Comparing the crystal structures of the R and S enantiomers, it was evident that the only functional group occupying the same position is the pyridine ring located in the S1 pocket (Figure S14E–G). The S-enantiomer is mainly anchored there to the binding site; moreover, the 2Fo–Fc density is less clear for this enantiomer, and its refined B-factors are higher, overall confirming the biochemical data obtained.

### 3.5 **X77**/Mpro in Aqueous Solution

Here we use MD to investigate the structural changes of three **X77**/Mpro complex structures (PDBids 8P57, 7PHZ, 6W63) solved in different saturation conditions and space groups, on passing from the solid state to the aqueous solution. Specifically, we perform 500-ns-long AMBER-based molecular dynamics simulations in explicit solvents of these systems. The Mpro structure and ligand pose remain stable during 500 ns of unbiased simulations for all of the three simulated systems (see Figure S15). The number of contacts between the two subunits is conserved for the systems with both cavities occupied (7PHZ, 6W63, Figure S16), independently of the space group, while for the single-cavity occupied system, this number increases, tightening up the subunit-to-subunit interaction (Figure S16).

**Figure 5:**
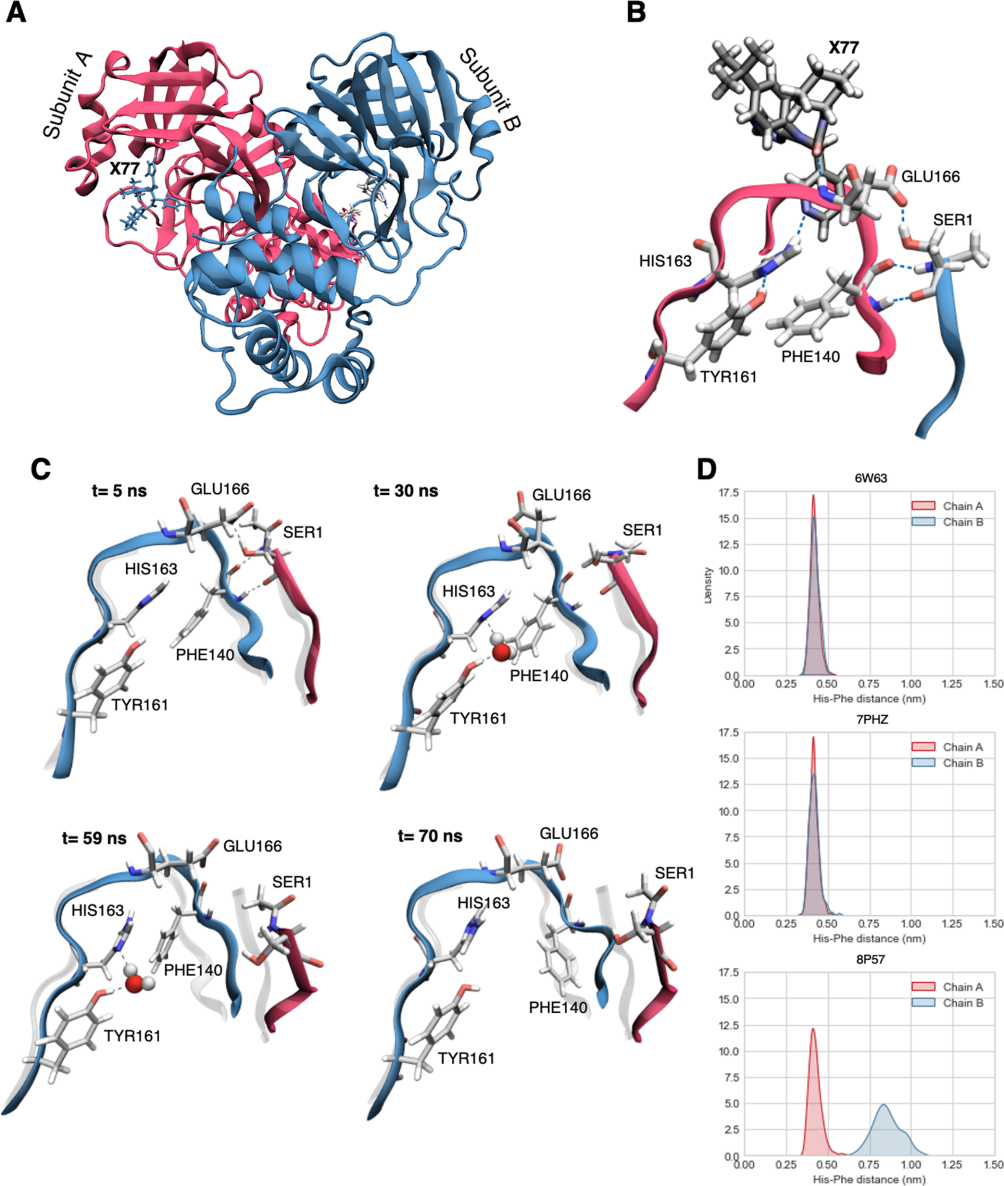
MD of **X77**/protein complexes in water solutions. (A) Cartoon representation of Mpro structure (in red, subunit A; in blue, subunit B; ligand **X77** is represented in blue sticks). (B) Hydrogen bond network in the binding site of subunit A in 8P57 after 5 ns of simulation. (C) Symmetry breaking happening at the level of the active site of subunit B in the unbiased simulation of 8P57. Residues that are relevant to the process are represented with gray sticks. One water molecule enters the binding site forming a bridge between HIS163 and TYR161. When the molecule exits the binding site, the hydrophobic contact between HIS163 and PHE140 is broken and the binding site inactivated. (D) Distribution of the distance between HIS163 and PHE140 rings during the last 400 ns of simulation of 6W63, 7PHZ, and 8P57.

**Figure 6:**
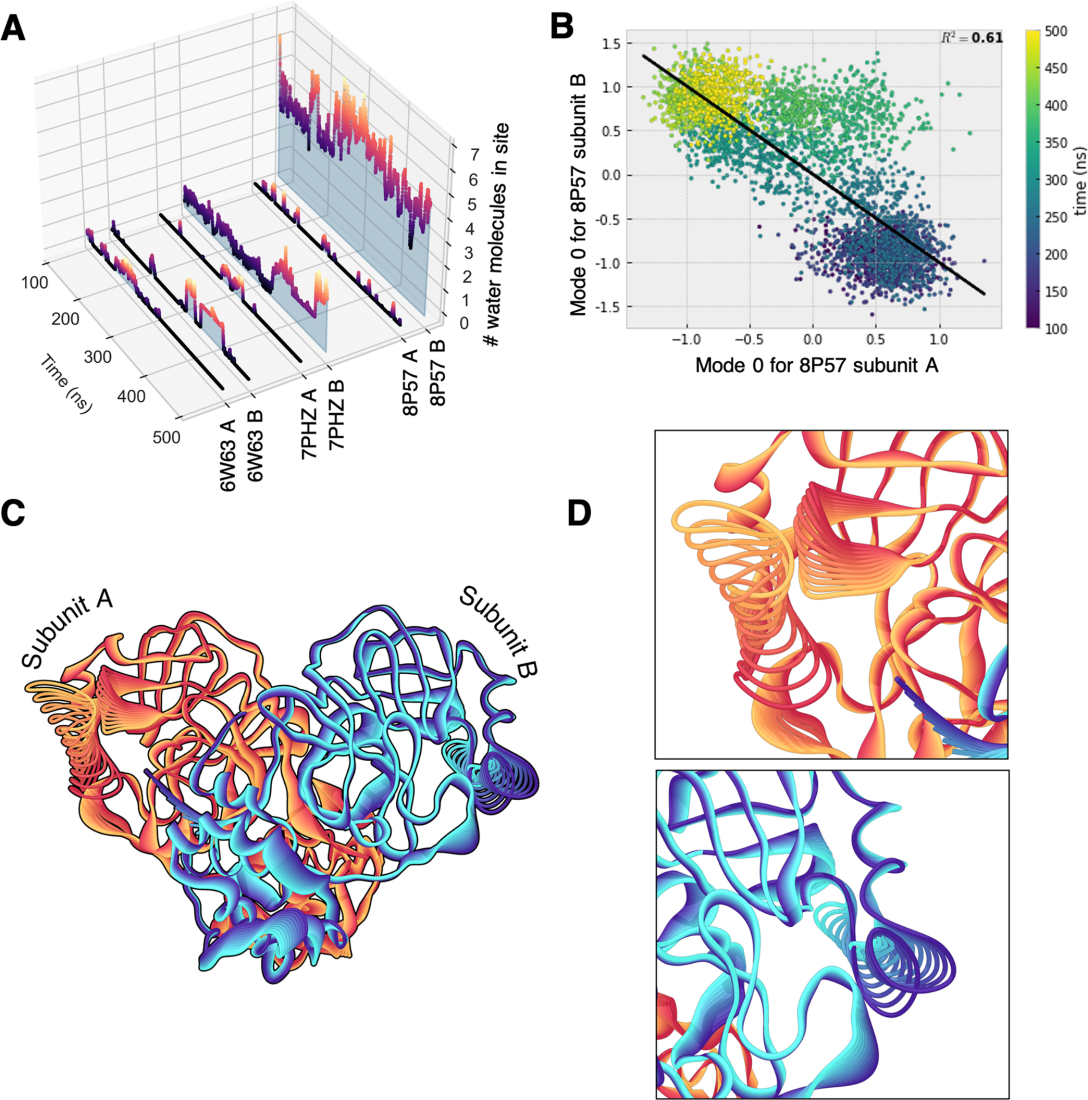
(A) Number of water molecules in the binding sites in subunits A and B of 6W63, 7PHZ, and 8P57 during our 500 ns of MD simulation. (B–D) PCA results for the singly occupied protein complex (8P57): (B) Values of the trajectories of subunits A and B projected on the eigenvector of the first principal component and their correlation; the analysis was performed also for the double-occupied enzymes, but no clear correlation was found (see Figure S18). (C) Structure of the protein deformed along the first eigenvector of the first principal component and (D) details of the binding sites. Subunits A and B are colored with a gradient from yellow to dark red and from dark blue to cyan, respectively. The gradient from a light to a dark color is inverted in subunit B to reflect the anticorrelation shown in panel (B).

To understand how solvation can impact on the catalytic site, we next define an ‘active’ geometry: this features the PHE140/HIS163 intrasubunit hydrophobic contact and the intersubunit interactions between the m-shaped loop and the N-finger of the adjacent subunit (Figure 5A,B).^35,54,55^ Such hydrophobic contacts of PHE140/HIS163 are analyzed in terms of the distance between the centers of these two rings as a function of time (*d*_CM_). The empty binding cavity (subunit B of 8P57) becomes inactive after a short simulation time (Figure 5C): *d*_CM_ passes from 0.46 (SD = 0.13) to 0.84 (SD = 0.08) nm. This is not the case for all of the other occupied cavities, where *d*_CM_ is 0.42 nm (SD ≤ 0.03) for the overall 500 ns of MD (Figure 5D). This suggests that, in the singly occupied protein, the presence of one ligand in one subunit might induce a nonactive geometry in the empty cavity of the adjacent subunit. Water plays a key role for this distortion: while basically absent in the occupied cavity (total number 0.07 (SD = 0.26)), as many as 4.29 (SD = 1.19) are present in the empty cavity (Figure 6A). As a result, HIS163 and PHE140 pi-pi stacking is broken, leading to an inactive state (Figure 5C,D). A principal component analysis (PCA) on each subunit further shows that the largest scale motion of subunit A is anticorrelated to that of subunit B: the former causes the closing, and the latter causes the opening of the binding cavity (Figure 6B–D). Interestingly, the trend of water occupation is also observed in the fully occupied enzymes. In subunit A, they have 0.12 (SD = 0.34) and 0.08 (SD = 0.26) number of water molecules, respectively, and in subunit B, 0.28 (SD = 0.49) and 0.91 (SD = 0.83), respectively. Notably, toward the end of the simulations, both subunits A are without water molecules, while both subunits B are with two water molecules on average (Figure 6A). This is more clear-cut in the asymmetric space group crystals (PDBid 7PHZ).

Next, we considered that the m-shaped loop of one subunit and the N-finger of the adjacent subunit interact via hydrogen bonds: i.e., GLU166 and PHE140 of one subunit and SER1 of the other. Such hydrogen bonds are only preserved in the fully symmetric double-occupied enzyme (symmetric space group), while they break for both subunits in the asymmetric space groups (P212121), either single or double occupied. These results suggest that symmetry might impact on the stability of such interaction and, in turn, of the ‘active’ geometry (Figure S17). However, such observation should be taken with care, since the highly flexible structure of the N-term plus the presence of artificial capping (see Section 2) might impact significantly on its dynamic behavior.

In conclusion, we observe a concerted opening of one site while closing off the other one in the single-occupied protein. In addition, the cavity that is not occupied (in subunit B) is highly hydrated in contrast to the other one. This latter trend is also observed (albeit to a lesser extent) for the doubly occupied enzymes.

### 3.6 A comment on saturating conditions

S-**X77** (Chart S1) does not exert any inhibitory activity at concentration 20 μM or lower (Figure 2). Strikingly, however, in excess concentration, it does bind the enzyme. The MX structure of the adduct has been solved here, and it shows double occupancy (see Supporting Information). This may be caused by the well-known high flexibility of the active site cavities,^33,56^ which allows the distorted second binding site to eventually accommodate the ligand in saturating condition. We conclude that Mpro can bind inhibitors if added in excess, forming doubly occupied adducts, even if the ligands exhibit no inhibitory activity.

## 4 Conclusions

Here, we have shown that X-ray structures at almost equimolar quantities of noncovalent and covalent ligands such as **X77** and **MG-132** (Chart 1) show only one active site occupied. The same asymmetry can be observed by leaving crystals of the doubly occupied enzyme in the drops for at least 2 months. Our MD simulations suggest that the single-occupied protein undergoes a further breaking of symmetry^32^ on passing from the solid state to solution. Water molecules enter the cavity and destabilize the PHE140-HIS163 contact (Figure 5) and, consequently, the catalytically active conformation of the HIS41/CYS145 dyad. This water-occupancy trend is also observed in the doubly occupied enzymes, although to a lesser extent. A similar, water-triggered breaking of symmetry in solution had been observed also for the apoprotein.^32,33^ The observed destabilization is associated with anticorrelated motions of the two subunits that close up the occupied binding cavity, while opening up the empty one (Figure 6B–D). This impacts the catalytic activity of the empty cavity, as the occupation of the binding cavity of one Mpro subunit by **X77** causes the loss of the catalytically active conformation in the other one. This observation, along with the MX results, suggests that the unoccupied chain in the formed dimer has a reduced affinity for a second ligand.

Taken together, our results strongly suggest that NC is operative for this enzyme. This would lead to two advantages in the ifecycle of the virus. First, it favors the ability to respond to a very wide range of ligand concentrations,^57^ making it very adaptable to the highly diverse local environments encountered by the enzyme during viral infection. Second, it allows the enzyme not to stop in saturation conditions.^9^ Such features may contribute to the ability of viral enzymes to function in different hosts’ conditions and, in turn, for virus survival and quickly adaptability to the host’s immune response and drug treatment. Our findings have significant implications for identifying effective inhibitors targeting Mpro, as well as other viral enzymes of the same family. Furthermore, the finding that noninhibiting molecules can still bind to the enzyme’s active sites emphasizes the importance of selecting appropriate reference compounds for ligand-based screening. Considering the structural asymmetry between the enzyme’s binding sites is also crucial for precise drug design by using structure-based methods. It is noteworthy that the virtual screening efficiency may vary between the two binding sites, necessitating careful consideration of their unique characteristics.^33^ Asymmetry emerges as a relevant theme in comprehending protein dynamics, particularly in the context of binding and reaction processes. This observation aligns with reports on the role of asymmetry in the behavior of numerous other dimers over the past decade, including Mitochondrial Hsp90 (TRAP1), phosphagen kinase, *Escherichia coli* TrpRS, and others.^58−69^ Furthermore, our study highlights the untapped potential of targeting the enzyme’s dimerization interface, an area with limited exploration for this enzyme class.^70^ Exploring this avenue allows for a broader range of ligands and holds great promise for advancing drug development strategies.^11^

It is also worthy to note that our MX results contrast with that found so far in the 500 MX structures, which exhibit double occupation and, in most cases (>98%), cyclic symmetry. This suggests that these studies probably were conducted with 2:1 ligand/monomer stoichiometry or more. The ligands, if in excess, may not be an inhibitor of Mpro even if they form doubly occupied adducts. Indeed, while **X77**—an R-enantiomer structure—inhibits Mpro in the μM-high nM range (Figure 2), the correspondent S-enantiomer (S-**X77**) does not exert any inhibitory activity at concentration 20 μM or lower (Figure 2). In excess concentration, however, it does bind the enzyme, as shown by the X-ray structure of the S-enantiomer/Mpro adduct (see Supporting Information). This may be caused by the well-known high flexibility of the active site cavities,^33,56^ which allows the distorted second binding site to eventually accommodate the substrate in saturating condition.

## Data Availability

Data, including input and parameter files for Molecular Dynamics simulations and scripts for water analysis can be found at Zenodo repository: 10.5281/zenodo.8366119.

## Supporting Information Available

Supporting Information about MX structures, MD simulation analysis and chemical synthesis (PDF)

## Abbreviations

SARS: severe acute respiratory syndrome
Mpro: main protease
MX: macromolecular crystallography
MD: molecular dynamics
PC: positive cooperativity
NC: negative cooperativity
