# Supplementary material for: Unexpected Single-Ligand Occupancy and Negative Cooperativity in the SARS-CoV-2 Main Protease
Source: J Chem Inf Model. 2023 Dec 5;64(3):892–904. doi: 10.1021/acs.jcim.3c01497 (PMC10865365; doi:10.1021/acs.jcim.3c01497)
Supplement: Supplementary file 1 — ci3c01497_si_001.pdf [file ci3c01497_si_001.pdf]

Supporting information: Unexpected single ligand occupancy and negative cooperativity in the SARS-CoV-2 Main protease

Simone Albani [‡,1,2], Elisa Costanzi [‡,3,†], Gia Linh Hoang [‡,4], Maria Kuzikov[5,6], Marcus Frings[7], Narjes Ansari[8], Nicola Demitri[3], Toan T. Nguyen[9], Valerio Rizzi[10], Jörg B. Schulz[4,11], Carsten Bolm[7], Andrea Zaliani[5,6], Paolo Carloni[*1,4,9], Paola Storici[*3], Giulia Rossetti[*1,4,11,12].

[‡] shared first authorship

[1] Institute for Neuroscience and Medicine (INM-9), Forschungszentrum Jülich, Jülich, 52425, Germany

[2] Faculty of Mathematics, Computer Science and Natural Sciences, RWTH Aachen, Aachen, 52062, Germany

[3] Elettra - Sincrotrone Trieste S.C.p.A., SS 14 - km 163, 5 in AREA Science Park, Trieste, Basovizza, 34149, Italy

[4] JARA-Brain Institute Molecular Neuroscience and Neuroimaging, Research Center Jülich GmbH, Jülich, 52425, and RWTH Aachen University, Aachen, 52056, Germany

[5] Fraunhofer Institute for Translational Medicine and Pharmacology (ITMP), Schnackenburgallee 114, 22525 Hamburg, Germany

[6] Fraunhofer Cluster of Excellence for Immune-Mediated Diseases (CIMD), Theodor Stern Kai 7, 60590 Frankfurt, Germany

[7] Institute of Organic Chemistry, RWTH Aachen University, Landoltweg 1, 52074 Aachen, Germany

[8] Atomistic Simulations, Italian Institute of Technology, Via Enrico Melen, 83, 16152, Genova, Italy

[9] Key Laboratory for Multiscale Simulation of Complex Systems, and Department of Theoretical Physics, Faculty of Physics, University of Science, Vietnam National University - Hanoi, 334 Nguyen Trai street, Thanh Xuan, Hanoi 11400, Vietnam

[10] School of Pharmaceutical Sciences, University of Geneva, Rue Michel Servet 1, 1206, Genève, Switzerland

[11] Department of Neurology, Medical Faculty, RWTH Aachen University, Aachen, 52074, Germany

[12] Jülich Supercomputing center (JSC), Forschungszentrum Jülich, Jülich, 52425, Germany

†present address: Center for Structural Studies, Heinrich-Heine Universität Düsseldorf, Universitätsstraße 1, 40225 Düsseldorf, Germany.

* Email: p.carloni@fz-juelich.de, paola.storici@elettra.eu, and g.rossetti@fz-juelich.de

I. INFORMATION ABOUT CHEMICAL SYNTHESIS

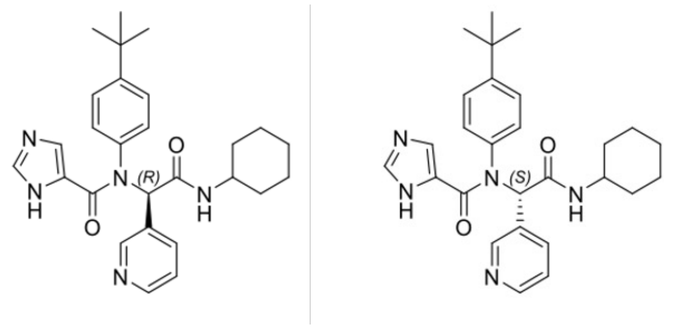

X77 **S-X77**

Chart S1. **X77** (R-enantiomer) and S-**X77** (S-enantiomer).

Commercially available chemicals were used as received. The supplier's specified assay of the reactants was accounted when the reaction batches were calculated.
For analytical thin layer chromatography (TLC) Merck's aluminum sheets silica gel 60 F_{254} were applied. Reaction compounds were visualized on TLC plates under ultraviolet light (λ = 254 nm) or by dipping them into an aqueous staining solution of potassium permanganate, potassium hydroxide and sodium carbonate. Then, the wet plates were dried with an electric heat gun. Flash column chromatography (FCC) was carried out with silica gel 60 M (0.040–0.063 mm) from Macherey-Nagel and an excess pressure (0.5 bar) of nitrogen. The quality of solvents for chromatography met technical grade and they were distilled prior to use. Mixing ratios of eluents for FCC and TLC were measured by volume.
Melting points (mp) were determined in open-end capillary tubes on a Büchi B-540 melting point apparatus.
Nuclear magnetic resonance (NMR) spectra were recorded at room temperature on an Agilent VNMR 600 (^{1}H NMR: 600 MHz, ^{13}C NMR: 151 MHz) spectrometer. All NMR data were manually processed and analyzed with MestReNova from Mestrelab Research. The chemical shifts δ are given in parts per million (ppm). Peak shifts in all ^{13}C NMR spectra were rounded to the nearest 0.1 ppm unless a greater precision was needed to distinguish closely spaced peaks. For ^{1}H NMR spectra the respective peak of the residual non-deuterated solvents ($CHCl_3$: δ_H = 7.26 ppm; CH_3OH: δ_H = 3.31 ppm) was used as reference. For ^{13}C NMR spectra the characteristic peak of the deuterated solvent ($CDCl_3$: δ_C = 77.16 ppm; CD_3OD: δ_C = 49.00 ppm) was taken as reference. All ^{13}C NMR data were generated with full decoupling of ^{1}H nuclei. Coupling patterns are described as br (broad), s (singlet), d (doublet), t (triplet), m (multiplet) or as combinations thereof (e.g., dd for doublet of doublets). Coupling constants J are reported in Hz. Data are listed in this style and order: "chemical shift (multiplicity, coupling constant, integration)".

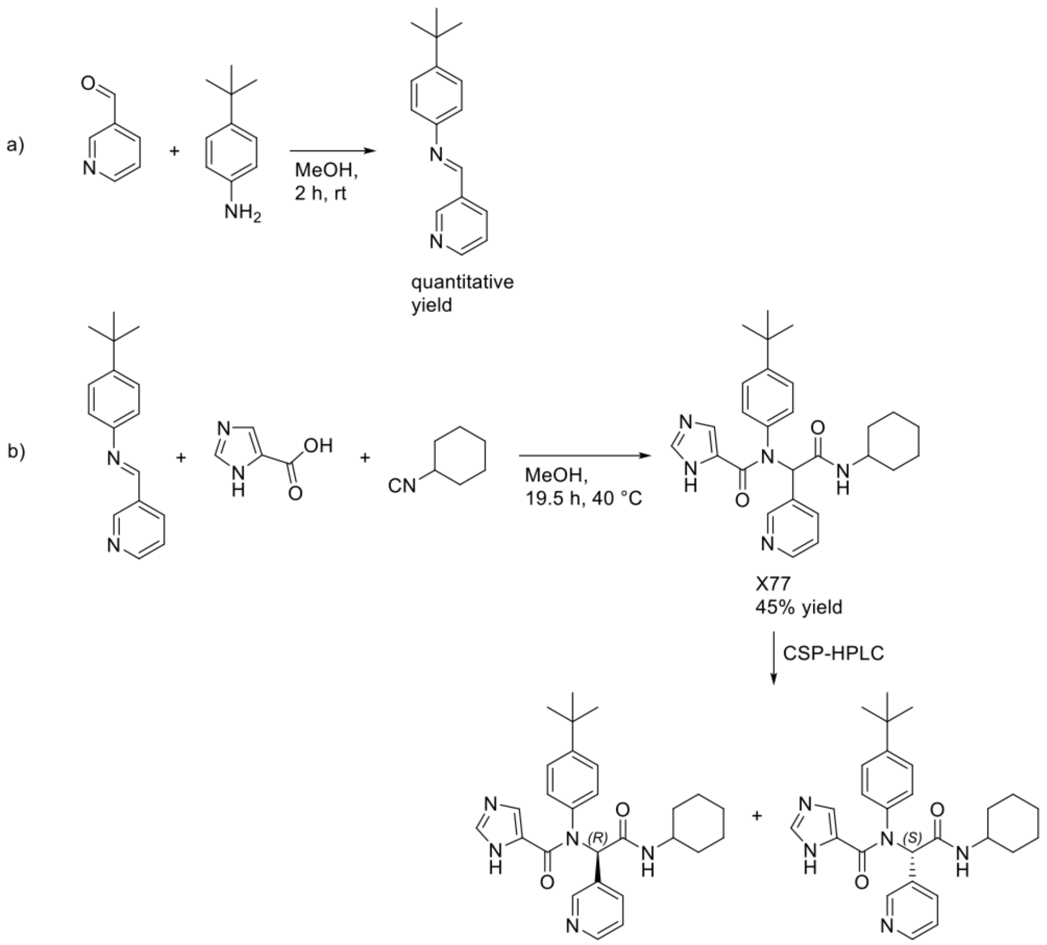

Scheme S1. a) Preparation of (E)-N-[4-(tert-butyl)phenyl]-1-(pyridin-3-yl)methanimine from 3-pyridinecarboxaldehyde and 4-(tert-butyl)aniline; b) synthesis of rac-X77 from (E)-N-[4-(tert-butyl)phenyl]-1-(pyridin-3-yl)methanimine, 4-imidazolecarboxylic acid and cyclohexyl isocyanide, followed by CSP-HPLC to isolate the (R)- and (S)-enantiomer.

Infrared (IR) spectra were recorded with attenuated total reflectance (ATR) technique on a PerkinElmer Spectrum 100 spectrometer with an attached UATR device Diamond KRS-5. Wave numbers ν of the absorptions are stated in cm^{-1}. All data were provided by the department of IR spectroscopy at the Institute of Organic Chemistry of RWTH Aachen University.
Regular mass (MS) spectra were acquired on a Finnigan SSQ 7000 spectrometer [electron ionization (EI): 70 eV; chemical ionization (CI): 100 eV, methane as reactant gas]. High-resolution mass (HRMS) spectra were recorded on a Thermo Scientific LTQ Orbitrap XL [electrospray ionization (ESI) in positive ion mode]. The relative mass accuracy Δ of the HRMS analysis is expressed in ppm and calculated on the basis of ppm = $10^6 \cdot (m_{observed} - m_{theory})/m_{theory}$. Peaks of characteristic fragmentation patterns are listed according to their m/z values. The percentage of intensity is given in parentheses. The data were retrieved from the department of MS spectrometry at the Institute of Organic Chemistry of RWTH Aachen University.
Elemental analyses (EA) were carried out on an Elementar Vario EL instrument and obtained from the microanalytical laboratory at the Institute of Organic Chemistry of RWTH Aachen University.
Analytical high-performance liquid chromatography (HPLC) measurements for the determination of enantiomers were conducted on an Agilent 1200-series with chiral stationary phase (CSP; length: 250 mm, diameter: 4.6 mm) from Chiral Technologies Inc. All generated HPLC data were processed with ChemStation from Agilent Technologies.

(*E*)-*N*-[4-(*tert*-Butyl)phenyl]-1-(pyridin-3-yl)methanimine

Mix 4-(*tert*-butyl)aniline (0.380 g, 2.55 mmol) and methanol (1 mL) in a round bottom flask at room temperature. Add 3-pyridinecarboxaldehyde (assay: 98%; 0.286 g, 2.62 mmol, 1.03 equiv). Stir reaction mixture continuously. Check progress by TLC. When conversion is complete (typically less than 2 h), remove methanol under reduced pressure at 50 °C. Dry the residual yellow oil in high vacuum at room temperature for several hours to yield the title compound.Typically, the product solidifies in high vacuum after a few minutes and is analytically pure.

Yield: 100%; **mp**: 60–61 °C (yellow solid); **^{1}H NMR** (600 MHz, $CDCl_3$): δ = 9.00 (s, 1H), 8.68 (d, J = 4.6 Hz, 1H), 8.51 (s, 1H), 8.28 (d, J = 7.7 Hz, 1H), 7.43 (d, J = 8.1 Hz, 2H), 7.39 (t, J = 6.5 Hz, 1H), 7.20 (d, J = 8.3 Hz, 2H), 1.34 (s, 9H) ppm; **^{13}C NMR** (151 MHz, $CDCl_3$): δ = 156.5, 151.9, 151.0, 149.9, 148.8, 134.9, 132.1, 126.2, 123.9, 120.7, 34.7, 31.5; **IR** (ATR): 2952, 2902, 2866, 2327, 2097, 1913, 1623, 1586, 1498, 1468, 1420, 1363, 1328, 1264, 1206, 1173, 1110, 1021, 979, 926, 881, 837, 705; **MS** (EI): 238 (77, $M^{+\bullet}$), 223 (100), 207 (12), 195 (13), 181 (12), 97 (13), 91 (10), 77 (10); **MS** (CI): 477 (34, $[2M + H]^+$), 461 (14, $[M + 223]^+$), 279 (7, $[M + C_3H_5]^+$), 267 (32, $[M + C_2H_5]^+$), 239 (100, $[M + H]^+$), 223 (8); **HRMS** (ESI): calculated for $C_{16}H_{19}N_2^+$ as $[M + H]^+$: m/z = 239.15428, found: m/z = 239.15477 with Δ = 2.07 ppm; **EA**: calculated for $C_{16}H_{18}N_2$ (239.34): C 80.63, H 7.61, N 11.75, found C 80.38, H 7.55, N 11.75.

The substance (*E*)-*N*-[4-(*tert*-Butyl)phenyl]-1-(pyridin-3-yl)methanimine has a CAS number (387865-82-9), but no experimental data are reported.

rac-*N*-[4-(*tert*-Butyl)phenyl]-*N*-[2-(cyclohexylamino)-2-oxo-1-(pyridin-3-yl)ethyl]-1*H*-imidazole-5-carboxamide (*rac*-X77)

Place first (*E*)-*N*-[4-(*tert*-butyl)phenyl]-1-(pyridin-3-yl)methanimine (209.6 mg, 0.8795 mmol), then 4-imidazolecarboxylic acid (assay: 98%; 103.5 mg, 0.9049 mmol, 1.03 equiv) and finally methanol (3 mL) into a sealable tube with screw cap. Close the tube and stir the mixture at 40 °C for 30 min. Open the tube, add cyclohexyl isocyanide (assay: 98%; 112 µL, 0.879 mmol, 1.00 equiv) and close the tube again. Continue stirring at 40 °C for 19 h. Eliminate methanol under reduced pressure at 50 °C. Purify the target compound by FCC and use dichloromethane/methanol/triethylamine = 375 : 25 : 2 as eluent mixture.

If the product is obtained as yellowish brown solid after FCC, suspend the colored solid in *n*-hexane. Add a small amount of chloroform and place the suspension into an ultrasonic bath for a few minutes. Carefully remove the supernatant solution and isolate the product as white solid.

Yield: 45%; **mp**: 157–158 °C (white solid); **^{1}H NMR** (600 MHz, CD_3OD): δ = 8.37 (d, J = 2.4 Hz, 1H), 8.33 (d, J = 5.0 Hz, 1H), 7.64–7.58 (m, 2H), 7.37–7.28 (m, 2H), 7.22 (dd, J = 7.8, 5.0 Hz, 1H), 6.28 (s, 1H), 5.44 (s br, 1H), 3.73–3.68 (m, 1H), 1.95–1.87 (m, 1H), 1.75 (dt, J = 13.9, 3.7 Hz, 2H), 1.68 (dt, J = 13.4, 4.1 Hz, 1H), 1.62 (dt, J = 13.0, 3.9 Hz, 1H), 1.42–1.06 (m, 17H); **^{13}C NMR** (151 MHz, CD_3OD): δ = 170.36, 153.96, 152.01, 149.64, 140.23, 137.87, 137.73, 132.94, 132.44, 127.13, 124.71, 64.16, 50.23, 35.56, 33.54, 33.51, 31.64, 26.60, 26.12, 26.03; **IR** (ATR): 3215, 3037, 2934, 2861, 2318, 2238, 2025, 1923, 1686, 1631, 1548, 1510, 1427, 1392, 1238, 1204, 1131, 1107, 1024, 999, 928, 894, 760, 711, 659; **MS** (EI): 460 (2, $[M + H]^+$), 365 (8), 360 (12), 345 (24), 334 (40), 239 (100), 223 (19), 95 (7), 56 (10); **MS** (CI): 460 (27, $[M + H]^+$), 361 (13), 272 (16), 244 (100), 228 (9), 219 (77), 188 (11); **HRMS** (ESI): calculated for $C_{27}H_{34}N_5O_2^+$ as $[M + H]^+$: m/z = 460.27070, found: m/z = 460.26945 with Δ = -2.72 ppm; **analytical CSP-HPLC** (Chiralpak AD-H, conditions 1): t_r = 5.4 min [enantiomer 1 = (*R*)-X77], t_r = 10.3 min [enantiomer 2 = (*S*)-X77], *n*-heptane/ethanol/methanol = 65:30:5, 0.7 mL/min, λ = 254 nm, 20 °C; **analytical CSP-HPLC** (Chiralpak AD-H, conditions 2): t_r = 6.1 min [enantiomer 1 = (*R*)-X77], t_r = 8.9 min [enantiomer 2 = (*S*)-X77], *n*-heptane/ethanol = 50:50, 0.6 mL/min, λ = 254 nm, 20 °C.

The NMR data are consistent with reported data[1]. Two conditions were developed for separating the enantiomers of *rac*-X77 on an analytical AD-H column (vide supra). In order to isolate them individually by preparative CSP-HPLC, an AD column (length: 250 mm, diameter: 50 mm) was used with *n*-hexane/ethanol = 75 : 25 as eluent mixture and a flow rate of 40 mL/min.

II. NMR SPECTRA AND HPLC CHROMATOGRAMS

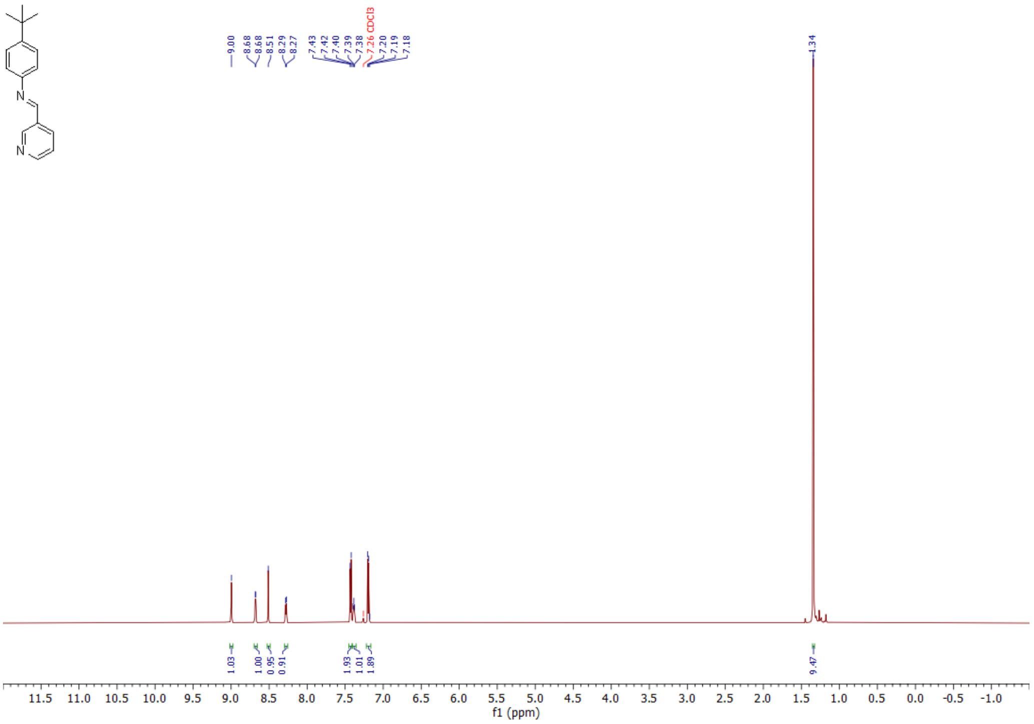

Figure S1. ^{1}H NMR of (*E*)-*N*-[4-(*tert*-butyl)phenyl]-1-(pyridin-3-yl)methanimine

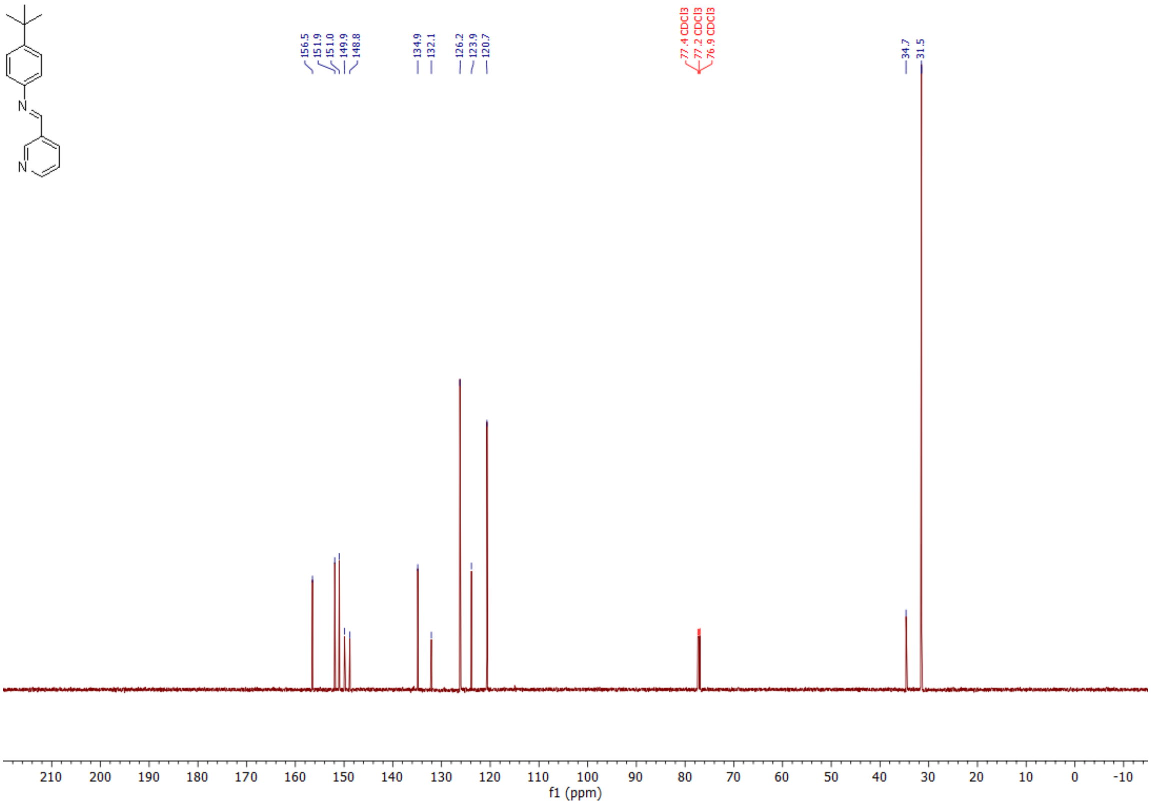

Figure S2. ^{13}C NMR of (*E*)-*N*-[4-(*tert*-butyl)phenyl]-1-(pyridin-3-yl)methanimine

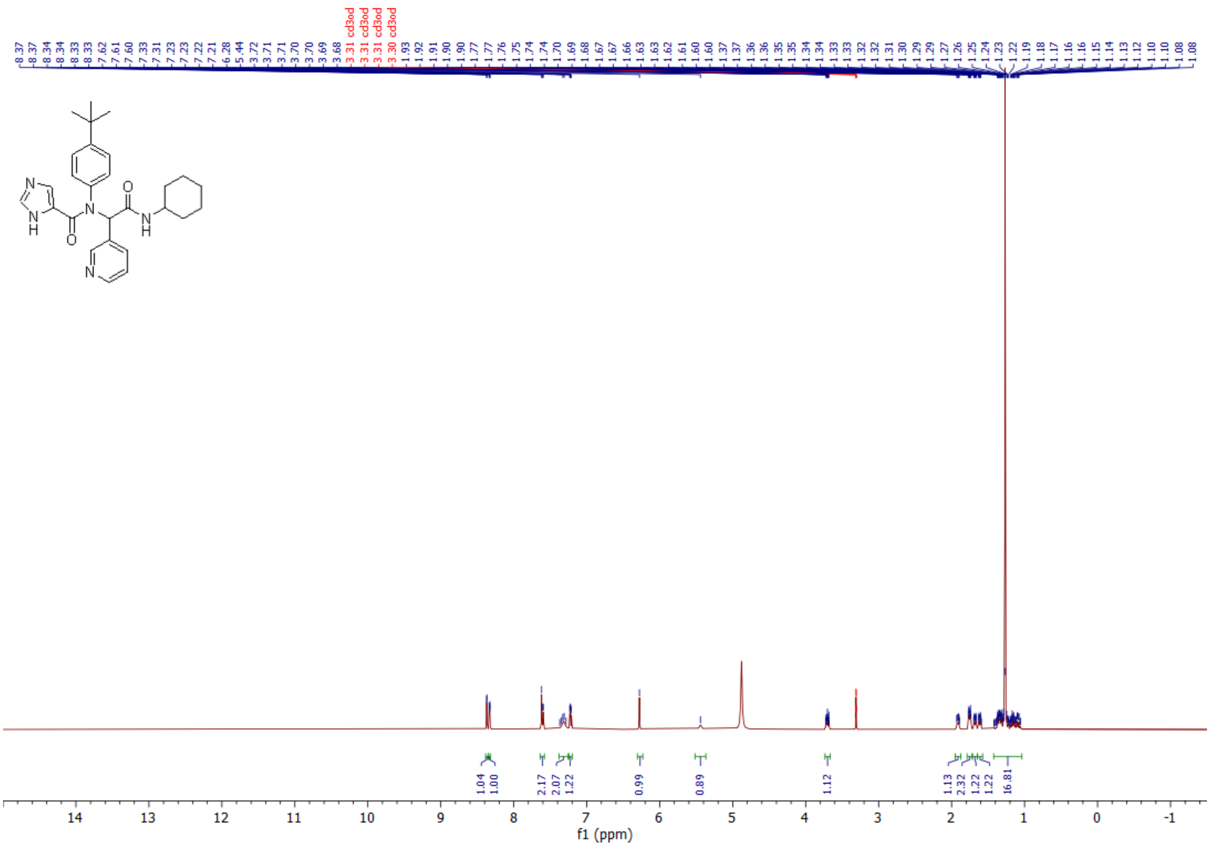

Figure S3. ^{1}H NMR of *rac*-*N*-[4-(*tert*-butyl)phenyl]-*N*-[2-(cyclohexylamino)-2-oxo-1- (pyridin-3-yl)ethyl]-1*H*-imidazole-5-carboxamide (*rac*-X77)

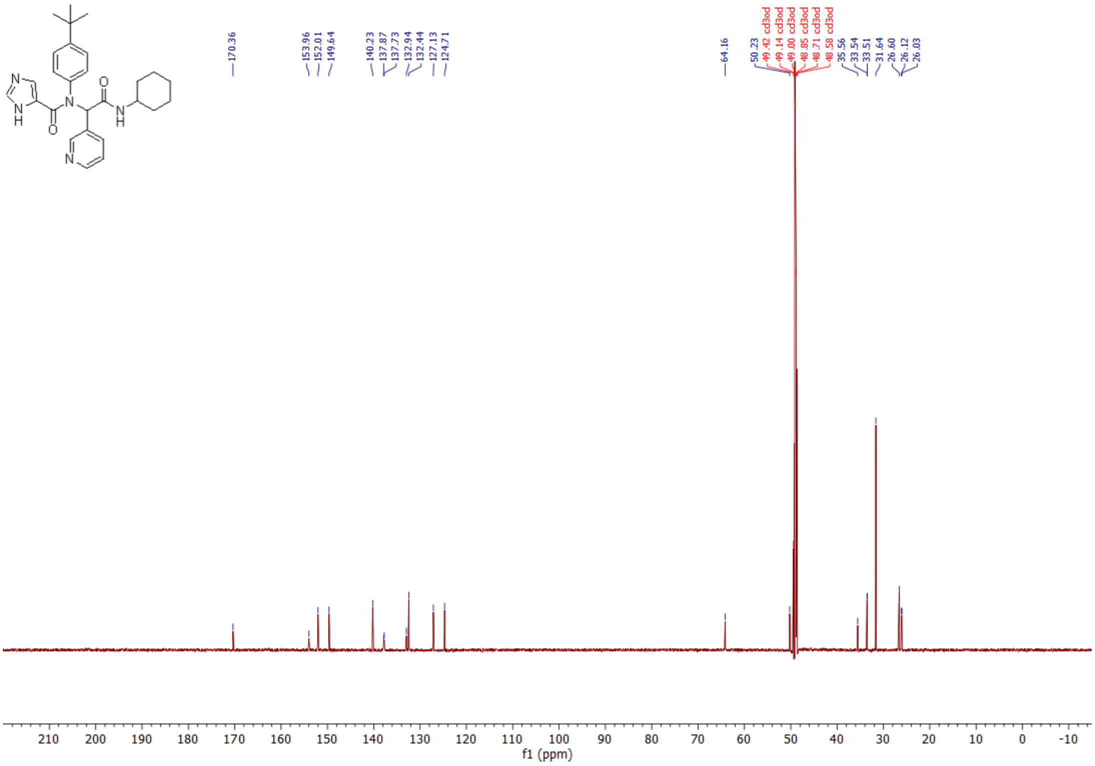

Figure S4. ^{13}C NMR of *rac*-*N*-[4-(*tert*-butyl)phenyl]-*N*-[2-(cyclohexylamino)-2-oxo-1- (pyridin-3-yl)ethyl]-1*H*-imidazole-5-carboxamide (*rac*-X77)

Sample Info: AD-H, Hep/EtOH/MeOH = 65:30:5, 0.7 mL/min, 20 °C

Instrument Conditions:	At Start	At Stop
Temperature in °C:	24.1	22.1
Pressure in bar:	63.3	63.2
Flow in mL/min:	0.70	0.70

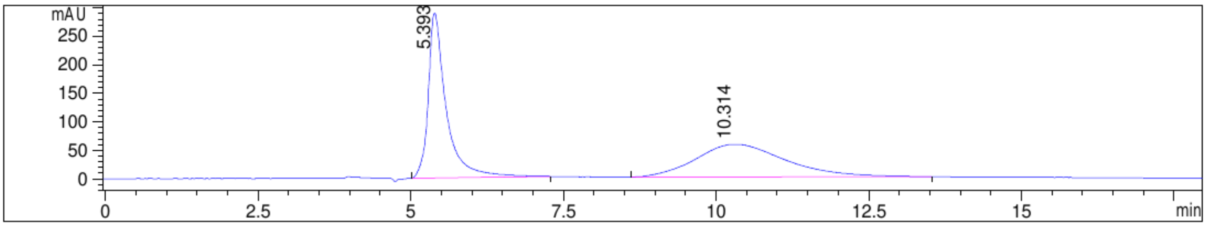

DAD1 A, Sig=254,4 Ref=360,100

Peak #	Ret. Time in min	Width in min	Height in mAU	Area in mAU*s	Area %
1	5.393	0.3420	289.23914	5935.88037	49.9890
2	10.314	1.7186	57.58877	5938.49072	50.0110
Total				11874.37109	100.0000

Figure S5. Analytical CSP-HPLC run with AD-H 1 conditions for *rac*-*N*-[4-(*tert*-butyl)phenyl]-*N*-[2-(cyclohexylamino)-2-oxo-1-(pyridin-3-yl)ethyl]-1*H*-imidazole-5-carboxamide (*rac*-X77)

Sample Info: AD-H, Hep/EtOH = 50:50, 0.6 mL/min, 20 °C

Instrument Conditions:	At Start	At Stop
Temperature in °C:	20.0	20.0
Pressure in bar:	65.1	65.7
Flow in mL/min:	0.60	0.60

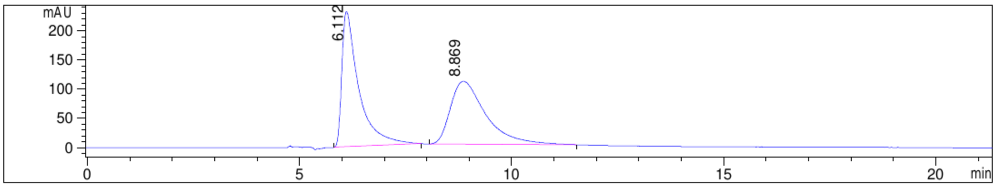

DAD1 A, Sig=254,4 Ref=360,100

Peak #	Ret. Time in min	Width in min	Height in mAU	Area in mAU*s	Area %
1	6.112	0.4622	231.51067	6419.69678	50.6128
2	8.869	0.9692	107.71679	6264.24658	49.3872
Total				12683.94336	100.0000

Figure S6. Analytical CSP-HPLC run with AD-H 2 conditions for *rac*-*N*-[4-(*tert*-butyl)phenyl]-*N*-[2-(cyclohexylamino)-2-oxo-1-(pyridin-3-yl)ethyl]-1*H*-imidazole-5-carboxamide (*rac*-X77)

Chromatogram : AK_Bo_MF-X77-rac_channel1

System : PREP
Method : varian-1
User : Varian
Run Time: 91.52

Acquired : 26.04.2021 09:58:00
Processed : 26.04.2021 11:21:07
Printed : 26.04.2021 13:17:17

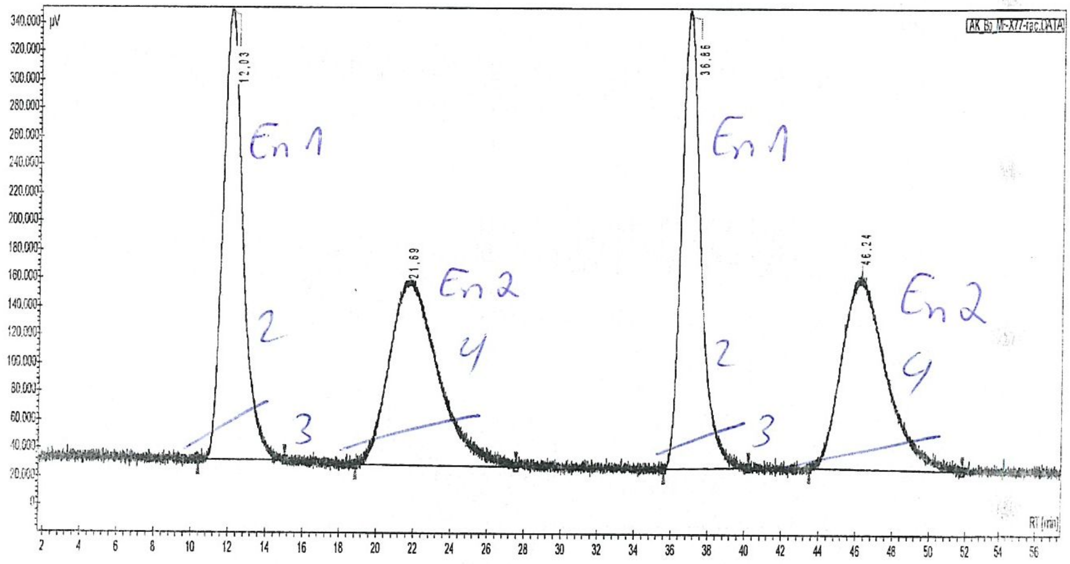

Peak results :

AK_Bo_MF-X77-rac.DATA [UV Kanal]

Index	Name	Time [Min]	Quantity [% Area]	Height [µV]	Area [µV.Min]	Area % [%]
1	UNKNOWN	12,03	25,16	317266,0	392129,3	25,155
2	UNKNOWN	21,69	25,41	128081,1	396096,5	25,410
3	UNKNOWN	36,86	25,07	319932,2	390774,1	25,068
4	UNKNOWN	46,24	24,37	140112,8	379835,1	24,367
Total			100,00	905392,1	1558835,1	100,000

Figure S7. Preparative CSP-HPLC run on AD column for *rac*-*N*-[4-(*tert*-butyl)phenyl]-*N*-[2-(cyclohexylamino)-2-oxo-1-(pyridin-3-yl)ethyl]-1*H*-imidazole-5-carboxamide (*rac*-X77)

Chromatogram : AKBo_MF-X77-rac-Frak2_ADH_7525_flow1_HexEtOH_30min4

Data file: AKBo_MF-X77-rac-Frak2_ADH_7525_flow1_HexEtOH_30min4.DATA
Method: HPLC1_ADH_7525_flow1_HexEtOHacq_30
Date: 26.04.2021 11:28:57

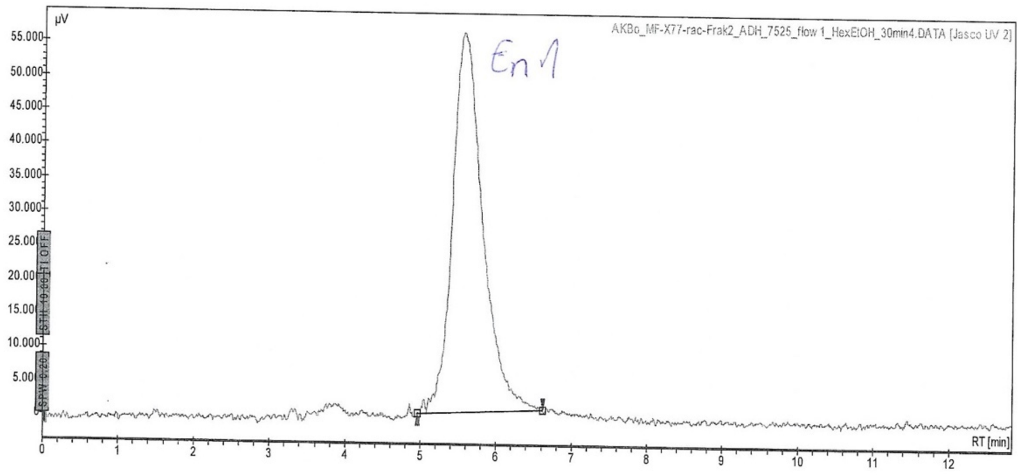

AKBo_MF-X77-rac-Frak2_ADH_7525_flow1_HexEtOH_30min4.DATA [Jasco UV 2]

Index	Start	Time	End	Area %
	[Min]	[Min]	[Min]	[%]
1	4,962	5,558	6,620	100,000
Total				100,000

Figure S8. Analytical CSP-HPLC run on AD-H column for (*R*)-*N*-[4-(*tert*-butyl)phenyl]-*N*-[2-(cyclohexylamino)-2-oxo-1-(pyridin-3-yl)ethyl]-1*H*-imidazole-5-carboxamide [(*R*)-X77, enantiomer 1] after separation

Chromatogram : AKBo_MF-X77-rac-Frak4_ADH_7525_flow1_HexEtOH_30min5

Data file: AKBo_MF-X77-rac-Frak4_ADH_7525_flow1_HexEtOH_30min5.DATA
Method: HPLC1_ADH_7525_flow1_HexEtOHacq_30
Date: 26.04.2021 11:44:13

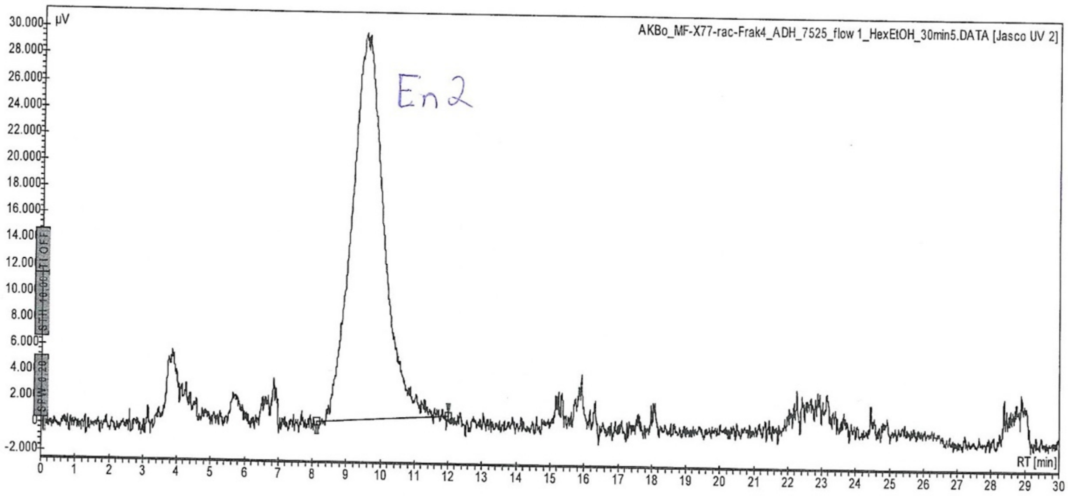

AKBo_MF-X77-rac-Frak4_ADH_7525_flow1_HexEtOH_30min5.DATA [Jasco UV 2]

Index	Start	Time	End	Area %
	[Min]	[Min]	[Min]	[%]
1	8,120	9,500	11,994	100,000
Total				100,000

Figure S9. Analytical CSP-HPLC run on AD-H column for (*S*)-*N*-[4-(*tert*-butyl)phenyl]-*N*-[2-(cyclohexylamino)-2-oxo-1-(pyridine-3-yl)ethyl]-1*H*-imidazole-5-carboxamide [(*S*)-X77, enantiomer 2] after separation

III. MACROMOLECULAR CRYSTALLOGRAPHY

Table S1. MX structures data collection and refinement statistics. Continued on Next Page.

	mPRO : X77 in $P2_12_12_1$	*mPRO : X77 75 µM*	*mPRO : X77 150 µM*	*mPRO : MG132 75 µM*	*mPRO : MG132 150 µM*
**Data collection**					
PDBid	7PHZ	8P54	8P55	8P56	8P57
Space group	$P2_12_12_1$	$P2_12_12_1$	$P2_12_12_1$	$P2_12_12_1$	$P2_12_12_1$
Unit cell parameters					
a, b, c (Å)	67.69, 100.58, 104.39	67.66, 100.83, 104.65	67.94, 101.01, 104.20	67.63, 100.70, 103.88	67.74, 99.72, 103.60
α, β, γ (°)	90.00, 90.00, 90.00	90.00, 90.00, 90.00	90.00, 90.00, 90.00	90.00, 90.00, 90.00	90.00, 90.00, 90.00
Cell volume (Å^3)	710692.191	713911.267	715095.250	707465.893	699841.797
Solvent content (%)	53.26	53.38	53.54	52.95	52.44
Matthews coefficient (Å^3/Da)	2.63	2.64	2.65	2.61	2.59
Wavelength (Å)	1.0000	0.999	0.999	0.999	0.999
Resolution (Å)	104.39-1.66 (1.69-1.66)	104.65 -1.60 (1.63-1.60)	104.02-1.63 (1.66-1.63)	67.63-1.85 (1.89-1.85)	71.85 -1.60 (1.63-1.60)
Number of unique reflections	84823 (4313)	95019 (4606)	90072 (4422)	60402 (3671)	93197 (4580)
R_{merge}	0.083 (1.474)	0.124 (1.575)	0.084 (1.488)	0.121 (0.797)	0.088 (1.478)
R_{meas}	0.087 (1.560)	0.131 (1.674)	0.089 (1.576)	0.137 (0.907)	0.093 (1.569)
R_{pim}	0.028 (0.505)	0.044 (0.562)	0.029 (0.514)	0.067 (0.425)	0.031 (0.522)
$\langle I/\sigma(I)\rangle$	15.8 (1.5)	9.8 (1.5)	14.0 (1.5)	7.1 (1.5)	13.8 (1.5)
$CC^{1/2}$	0.999 (0.690)	0.997 (0.581)	0.999 (0.613)	0.994 (0.713)	0.999 (0.618)
Completeness (%)	100.0 (100.0)	100.0 (100.0)	100.0 (100.0)	99.5 (99.2)	100.0 (100.0)
Multiplicity	9.1 (9.3)	8.8 (8.8)	9.0 (9.3)	4.2 (4.1)	8.8 (8.8)
**Refinement**					
Resolution (Å)	41.33 -1.66	49.50 -1.60	45.45-1.63	41.91-1.85	44.93-1.60
Number of reflections	84700	94917	89972	60314	93106
Number of reflections (R-Free)	4173	4838	4399	2931	4580
R_{work}/R_{free} (%)	16.12 / 18.15	16.75 / 18.33	16.25 / 18.40	16.63 / 20.09	16.62/18.60
r.m.s. deviations					
bond length (Å)	0.009	0.009	0.008	0.012	0.010
bond angles (°)	1.101	1.124	0.977	1.018	0.974
Ramachandran plot					
favored (%)	98.02	98.35	98.68	98.68	98.35
allowed (%)	1.82	1.65	1.32	1.32	1.65
outliers (%)	0.16	0.00	0.00	0.00	0.00

	mPRO : R-X77 500 µM	*mPRO : R-X77 5 mM*	*mPRO : S-X77 500 µM*	*mPRO : S-X77 5 mM*	*mPRO : MG132 "old"*	*mPRO : X77 "old"*
**Data collection**						
PDBid	8P58	8P5A	8P5B	8P5C	8P86	8P87
Space group	$P2_12_12_1$	$P2_12_12_1$	$P2_12_12_1$	$P2_12_12_1$	$P2_12_12_1$	$P2_12_12_1$
Unit cell parameters						
a, b, c (Å)	67.69, 100.83, 104.33	67.93, 99.83, 103.72	67.57, 100.25, 103.9	68.30, 99.86, 103.78	68.37, 99.35, 102.80	67.85, 101.14, 104.26
α, β, γ (°)	90.00, 90.00, 90.00	90.00, 90.00, 90.00	90.00, 90.00, 90.00	90.00, 90.00, 90.00	90.00, 90.00, 90.00	90.00, 90.00, 90.00
Cell volume (Å^3)	712029.323	703379.237	703894.234	707791.492	698248.084	698248.084
Solvent content (%)	53.25	52.68	52.71	52.97	53.64	53.72
Matthews coefficient (Å^3/Da)	2.63	2.60	2.60	2.62	2.65	2.66
Wavelength (Å)	0.9718	0.9718	0.9718	0.9718	1.0000	0.9999
Resolution (Å)	104.33-1.55 (1.58-1.55)	103.72 -1.47 (1.49-1.47)	103.92-1.66 (1.69-1.66)	103.78-1.51 (1.54-1.51)	102.80 -1.85 (1.89-1.85)	101.14-1.70 (1.73-1.70)
Number of unique reflections	104143 (5143)	121551 (5949)	83674 (4042)	111866 (5480)	60427 (3669)	79582 (4157)
R_{merge}	0.107 (1.604)	0.076 (1.577)	0.097 (1.629)	0.138 (1.880)	0.080 (1.310)	0.083 (1.525)
R_{meas}	0.112 (1.685)	0.080 (1.677)	0.102 (1.709)	0.144 (1.958)	0.088 (1.431)	0.088 (1.617)
R_{pim}	0.034 (0.512)	0.027 (0.565)	0.030 (0.507)	0.040 (0.544)	0.035 (0.571)	0.029 (0.531)
$\langle I/\sigma(I) \rangle$	12.5 (1.5)	13.9 (1.5)	14.0 (1.5)	10.0 (1.5)	12.6 (1.5)	14.8 (1.5)
$CC^{1/2}$	0.999 (0.653)	0.999 (0.664)	0.999 (0.661)	0.997 (0.594)	0.998 (0.594)	0.999 (0.660)
Completeness (%)	100.0 (100.0)	100.0 (100.0)	99.7 (99.1)	100.0 (100.0)	99.9 (100.0)	100.0 (100.0)
Multiplicity	10.8 (10.6)	8.9 (8.7)	11.0 (11.0)	12.7 (12.8)	6.3 (6.2)	9.1 (9.0)
**Refinement**						
Resolution (Å)	49.48 -1.55	51.86 -1.47	49.32-1.66	49.54-1.51	49.39 -1.85	50.57-1.70
Number of reflections	104034	121365	83577	111766	60345	79486
Number of reflections (R-Free)	5304	5993	4313	5760	2921	4021
R_{work}/R_{free} (%)	15.89 / 17.90	16.44 / 17.80	16.56 / 18.89	15.90 / 17.83	17.31 / 19.69	17.01 / 19.14
r.m.s. deviations						
bond length (Å)	0.012	0.006	0.009	0.012	0.012	0.009
bond angles (°)	1.183	0.933	1.153	1.071	1.143	0.931
Ramachandran plot						
favored (%)	98.35	98.52	98.34	98.35	97.97	98.35
allowed (%)	1.48	1.48	1.66	1.65	1.86	1.65
outliers (%)	0.16	0.00	0.00	0.00	0.17	0.00

IV. SUPPLEMENTARY FIGURES

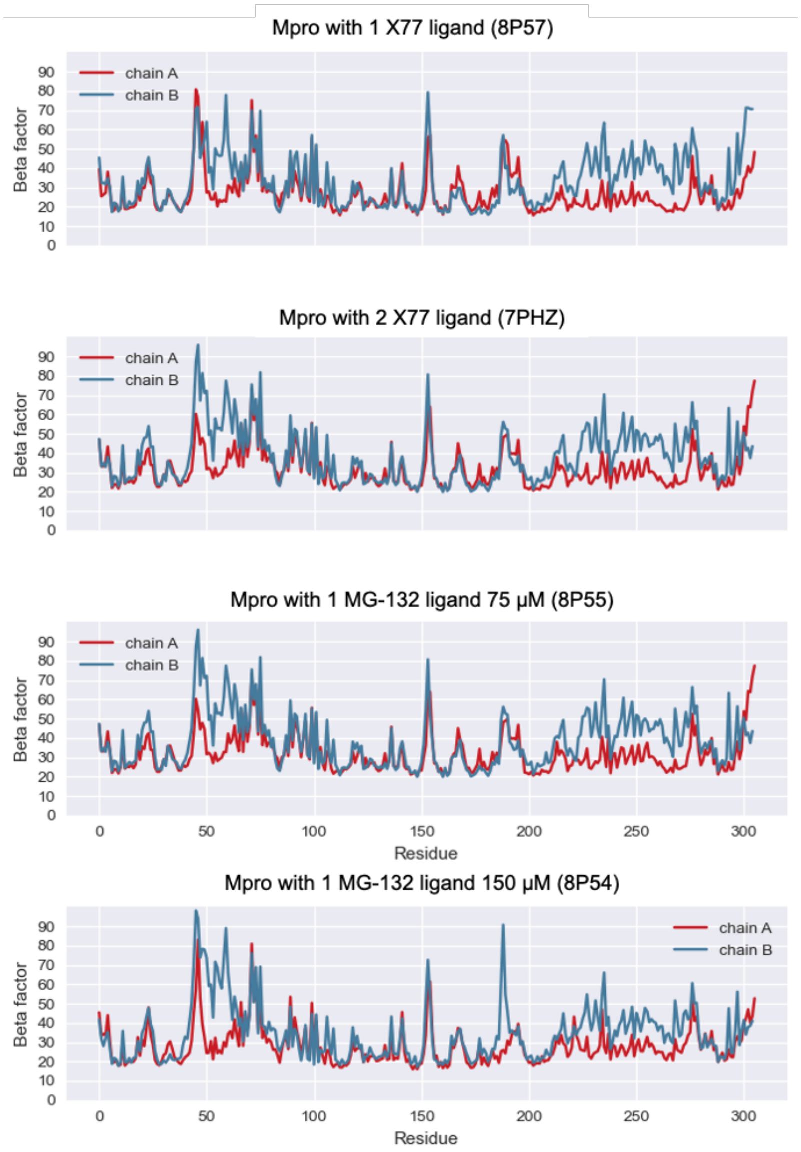

Figure S10. Comparison between average beta factor per residue in chain A vs. chain B in the structures with PDBid 8P57, 7PHZ, 8P55, and 8P54.

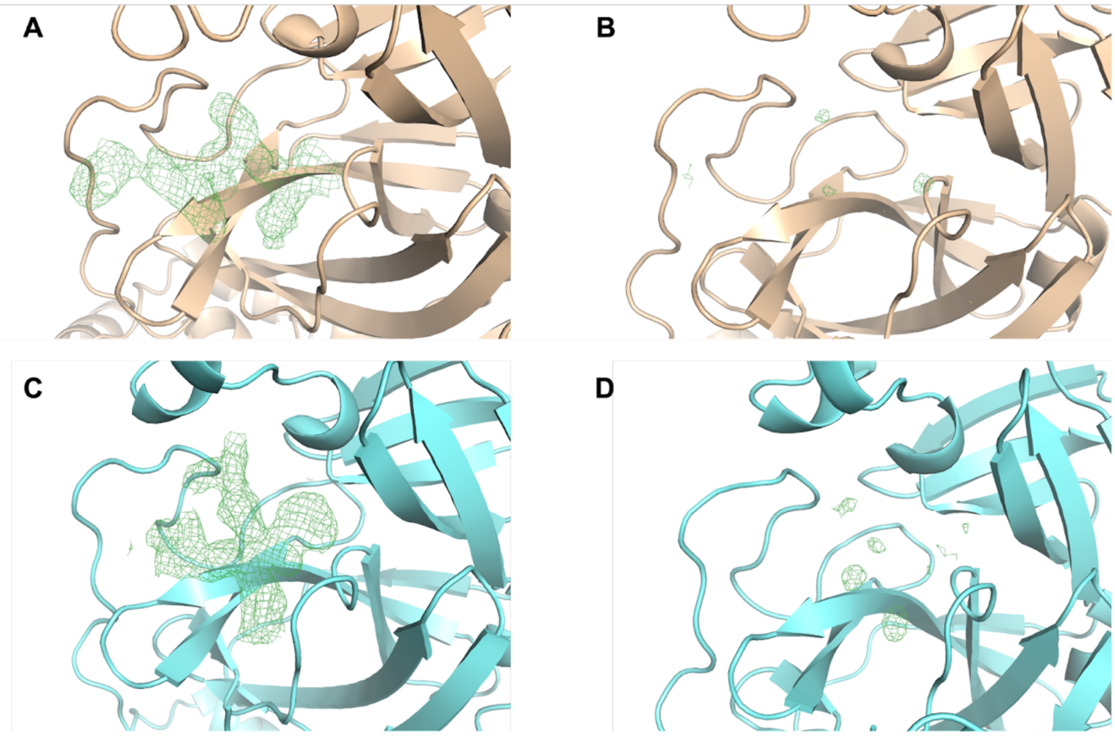

Figure S11 - Fo-Fc map contoured at 3 sigma is shown as green mesh **A.** MG132, chain A; **B.** MG132, chain B; **C.** X77, chain A, **D.** X77 chain B.

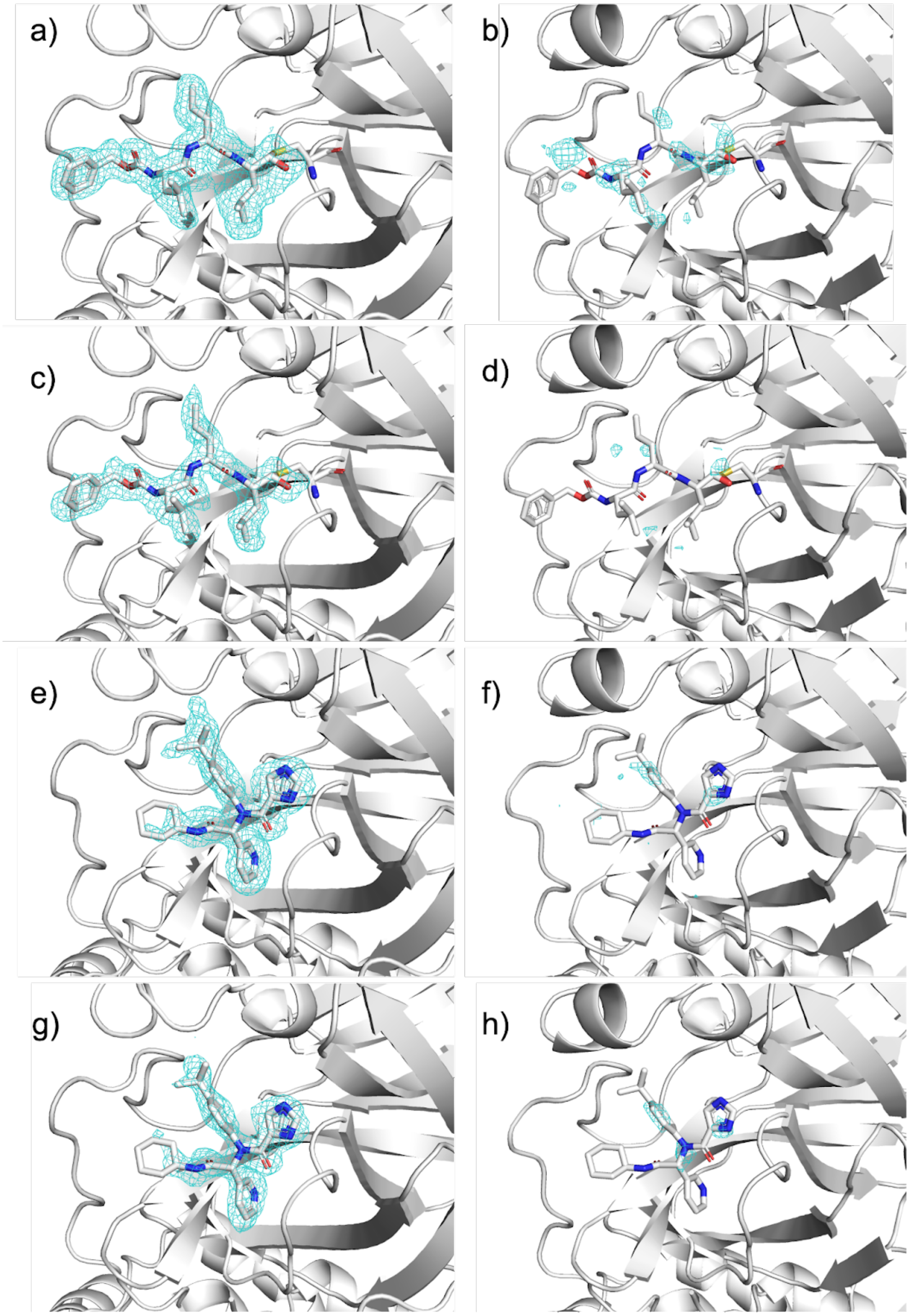

Figure S12. Polder omit maps are shown as mesh and contoured at 3 sigma. a) MG132 1:2 LMS, chain A; b) MG132 1:2 LMS, chain B; c) MG132 1:1 LMS, chain A; d) MG132 1:1 LMS, chain B; e) X77 1:2 LMS, chain A; f) X77 1:2 LMS, chain B; g) X77 1:1 LMS, chain A; h) X77 1:1 LMS, chain B.

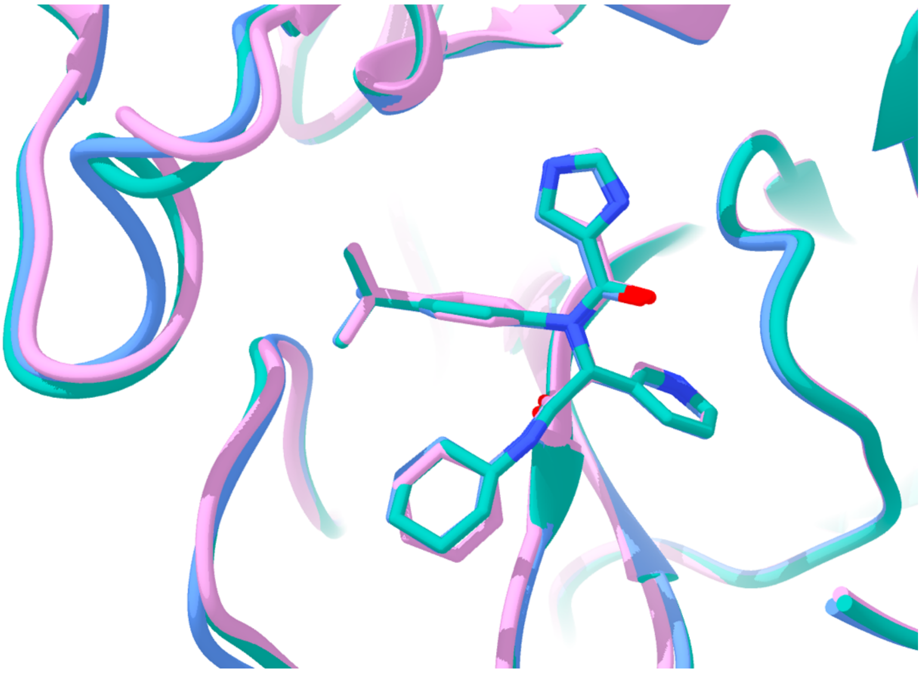

Figure S13. X77 in complex with Mpro (Chain A). The superimposed structures of PDBids 8P57 (sea green), 7PHZ (blue), and 6W63 (pink).

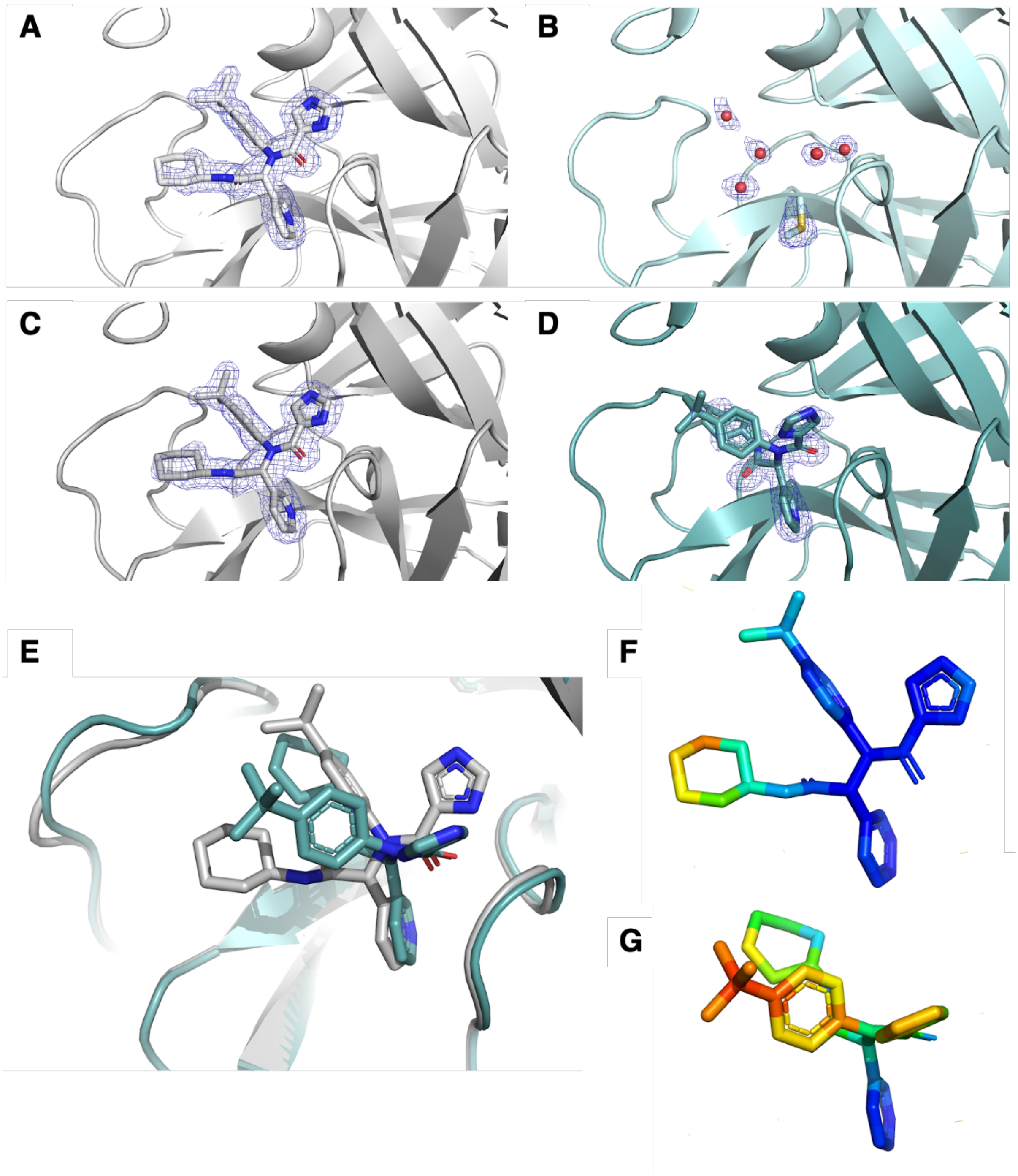

Figure S14 2 Fo-Fc map contoured at 1 sigma is shown as blue mesh. **A.** Enantiomer 1/R at 500 uM, **B.** Enantiomer 2/S at 500 uM, **C.** Enantiomer 1/R at 5 mM, **D.** Enantiomer 2/S at 5 mM. **E.** comparison of the binding poses of the R (gray) and S (teal) enantiomers. **F.** Enantiomer R colored by B factor **G.** Enantiomer S colored by B-factor.

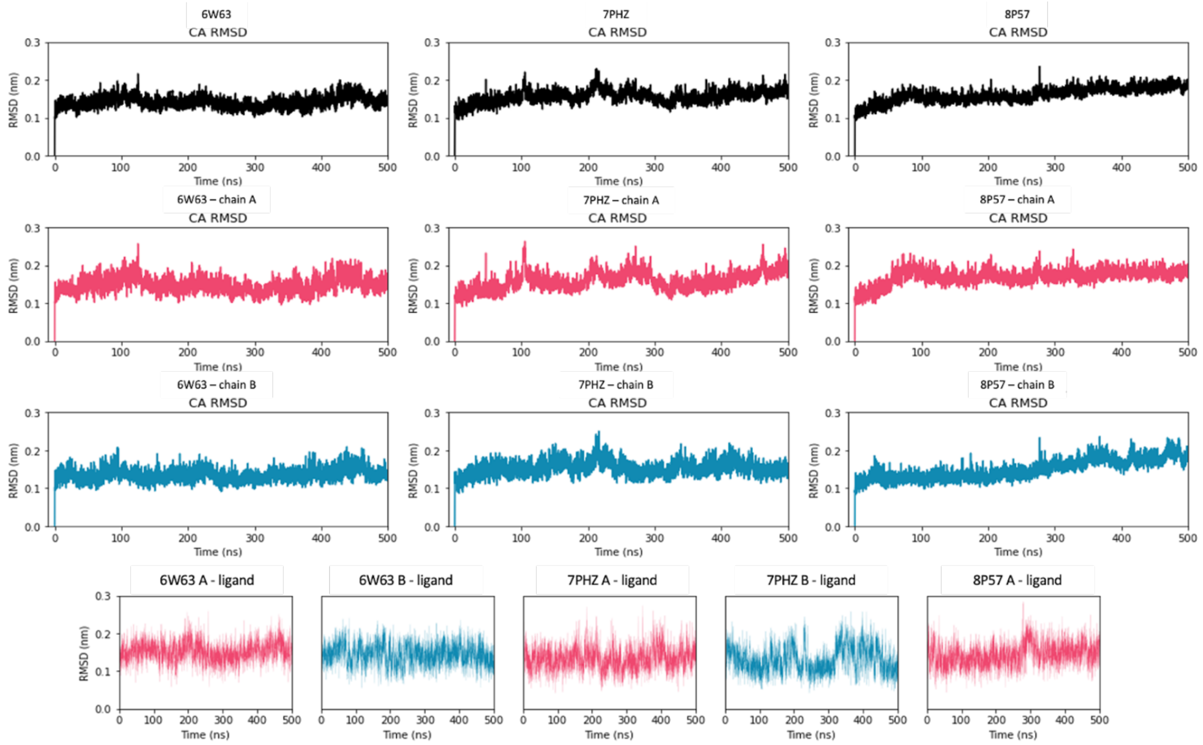

Figure S15. Root-mean-square deviation (RMSD) of Mpro during 500-ns MD simulation calculated over the CA atoms positions in the backbone after fitting. RMSD of **X77** during 500-ns unbiased simulations calculated over all non-hydrogen atoms.

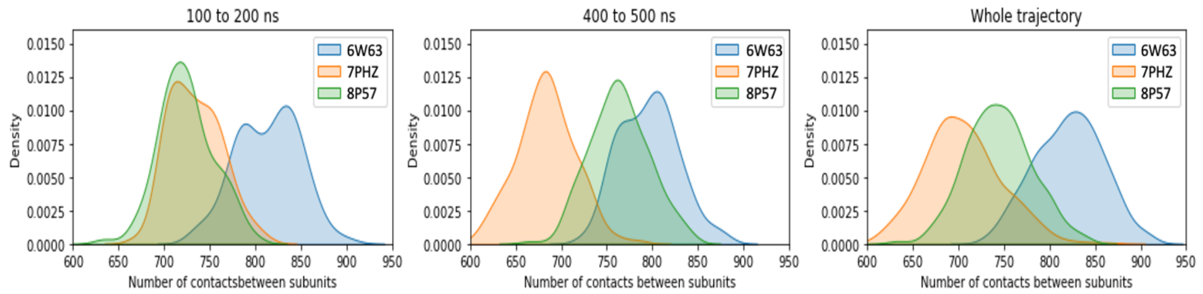

Figure S16. Number of contacts between subunit A and subunit B during the last 400 ns of unbiased MD simulation. The number was calculated using the COORDINATION function implemented in the open-source, community-developed PLUMED2 library[2]. Each subunit was split in different groups based on the atomic element and each group was analyzed in its interaction with the groups of the adjacent subunit using the sum of the Van Der Waals radii as reference distance R_0[3].

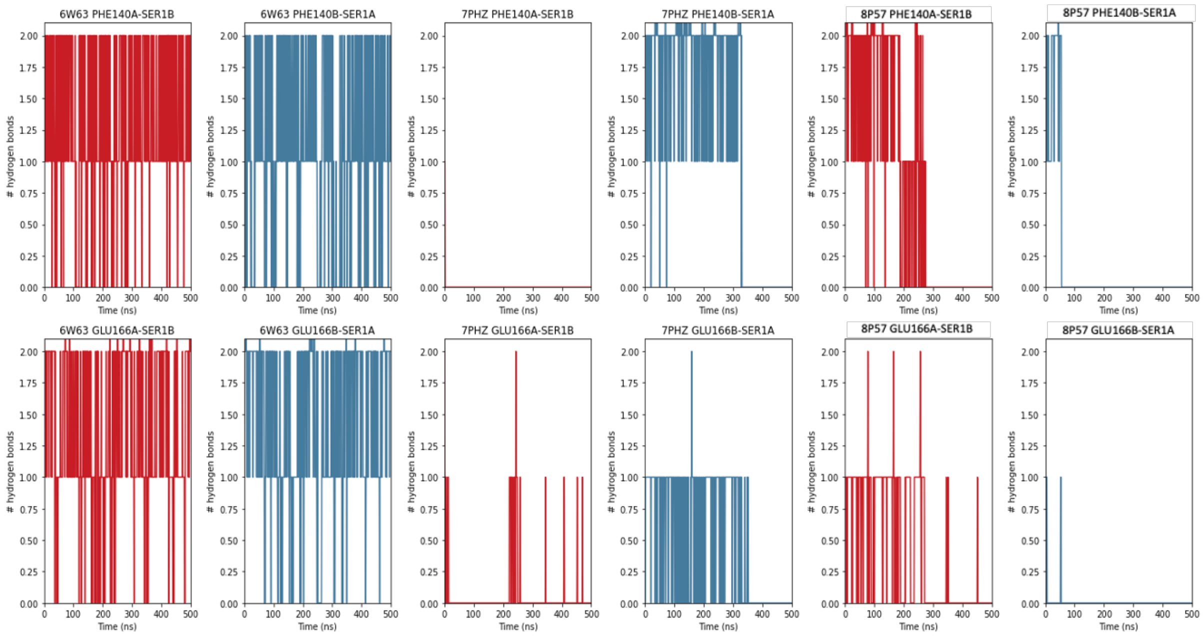

Figure S17. Number of hydrogen bonds between GLU166 and PHE140 of one subunit and SER1 of the adjacent subunit during 500-ns simulations.

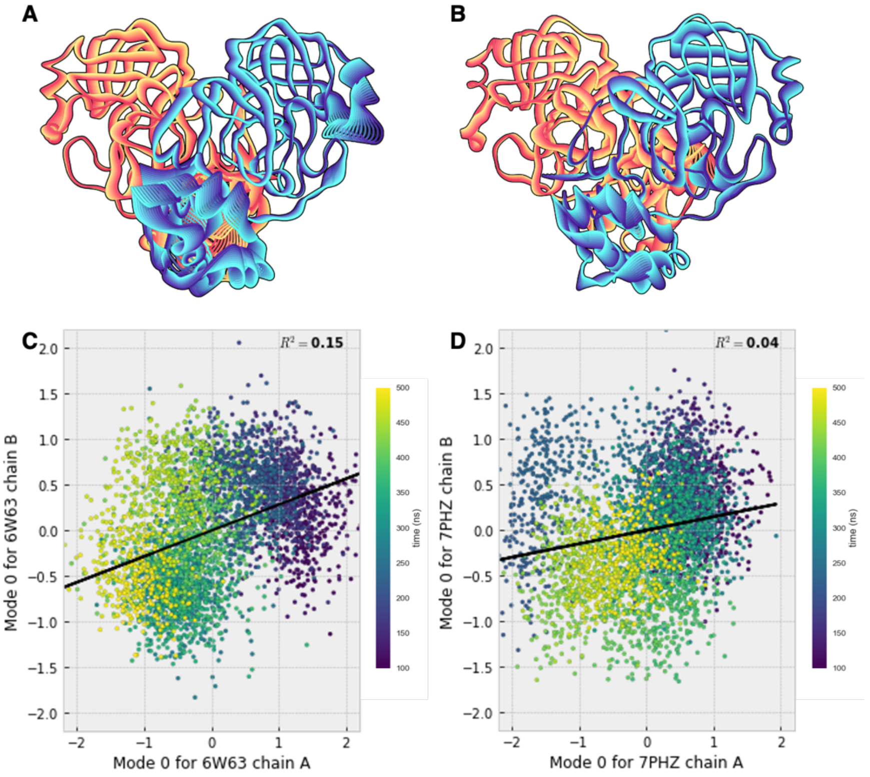

Figure S18. PCA results: Structure of 6W63 (**A**) and 7PHZ (**B**) deformed along the first eigenvector of their first principal component. Missing correlation between projections of the first principal component of subunit A and subunit B in the simulations of 6W63 (**C**) and 7PHZ (**D**).

REFERENCES

(1) Stille, J. K.; Tjutrins, J.; Wang, G.; Venegas, F. A.; Hennecker, C.; Rueda, A. M.; Sharon, I.; Blaine, N.; Miron, C. E.; Pinus, S.; Labarre, A.; Plescia, J.; Burai Patrascu, M.; Zhang, X.; Wahba, A. S.; Vlaho, D.; Huot, M. J.; Schmeing, T. M.; Mittermaier, A. K.; Moitessier, N. Design, Synthesis and in Vitro Evaluation of Novel SARS-CoV-2 3CLpro Covalent Inhibitors. *Eur. J. Med. Chem.* 2022, *229*, 114046. https://doi.org/10.1016/j.ejmech.2021.114046.

(2) Bonomi, M.; Branduardi, D.; Bussi, G.; Camilloni, C.; Provasi, D.; Raiteri, P.; Donadio, D.; Marinelli, F.; Pietrucci, F.; Broglia, R. A.; Parrinello, M. PLUMED: A Portable Plugin for Free-Energy Calculations with Molecular Dynamics. *Comput. Phys. Commun.* 2009, *180* (10), 1961–1972. https://doi.org/10.1016/j.cpc.2009.05.011.

(3) Bonomi, M.; Bussi, G.; Camilloni, C.; Tribello, G. A.; Banáš, P.; Barducci, A.; Bernetti, M.; Bolhuis, P. G.; Bottaro, S.; Branduardi, D.; Capelli, R.; Carloni, P.; Ceriotti, M.; Cesari, A.; Chen, H.; Chen, W.; Colizzi, F.; De, S.; De La Pierre, M.; Donadio, D.; Drobot, V.; Ensing, B.; Ferguson, A. L.; Filizola, M.; Fraser, J. S.; Fu, H.; Gasparotto, P.; Gervasio, F. L.; Giberti, F.; Gil-Ley, A.; Giorgino, T.; Heller, G. T.; Hocky, G. M.; Iannuzzi, M.; Invernizzi, M.; Jelfs, K. E.; Jussupow, A.; Kirilin, E.; Laio, A.; Limongelli, V.; Lindorff-Larsen, K.; Löhr, T.; Marinelli, F.; Martin-Samos, L.; Masetti, M.; Meyer, R.; Michaelides, A.; Molteni, C.; Morishita, T.; Nava, M.; Paissoni, C.; Papaleo, E.; Parrinello, M.; Pfaendtner, J.; Piaggi, P.; Piccini, G.; Pietropaolo, A.; Pietrucci, F.; Pipolo, S.; Provasi, D.; Quigley, D.; Raiteri, P.; Raniolo, S.; Rydzewski, J.; Salvalaglio, M.; Sosso, G. C.; Spiwok, V.; Šponer, J.; Swenson, D. W. H.; Tiwary, P.; Valsson, O.; Vendruscolo, M.; Voth, G. A.; White, A.; The PLUMED consortium. Promoting Transparency and Reproducibility in Enhanced Molecular Simulations. *Nat. Methods* 2019, *16* (8), 670–673. https://doi.org/10.1038/s41592-019-0506-8.
